# Identification of risk loci for primary aldosteronism in genome-wide association studies

**DOI:** 10.1038/s41467-022-32896-8

**Published:** 2022-09-03

**Authors:** Edith Le Floch, Teresa Cosentino, Casper K. Larsen, Felix Beuschlein, Martin Reincke, Laurence Amar, Gian-Paolo Rossi, Kelly De Sousa, Stéphanie Baron, Sophie Chantalat, Benjamin Saintpierre, Livia Lenzini, Arthur Frouin, Isabelle Giscos-Douriez, Matthis Ferey, Alaa B. Abdellatif, Tchao Meatchi, Jean-Philippe Empana, Xavier Jouven, Christian Gieger, Melanie Waldenberger, Annette Peters, Daniele Cusi, Erika Salvi, Pierre Meneton, Mathilde Touvier, Mélanie Deschasaux, Nathalie Druesne-Pecollo, Sheerazed Boulkroun, Fabio L. Fernandes-Rosa, Jean-François Deleuze, Xavier Jeunemaitre, Maria-Christina Zennaro

**Affiliations:** 1 Centre National de Recherche en Génomique Humaine, Institut de biologie François Jacob, CEA, Université Paris-Saclay, Evry, France; 2 Université Paris Cité, Inserm, PARCC, F-75015 Paris, France; 3 Medizinische Klinik und Poliklinik IV, Ludwig-Maximilians-University, 80336 Munich, Germany; 4 Klinik für Endokrinologie, Diabetologie und Klinische Ernährung, Universitätsspital Zürich (USZ) und Universität Zürich (UZH), Zürich, Switzerland; 5 Assistance Publique-Hôpitaux de Paris, Hôpital Européen Georges Pompidou, Unité Hypertension artérielle, Paris, France; 6 DMCS ‘G. Patrassi’ University of Padova Medical School, University Hospital, 35126 Padova, Italy; 7 Université Paris Cité, F-75006 Paris, France; 8 Assistance Publique-Hôpitaux de Paris, Hôpital Européen Georges Pompidou, Service de Physiologie, Paris, France; 9 Université Paris Cité, Institut Cochin, Genom’IC platform, INSERM, CNRS, 75014 Paris, France; 10 Assistance Publique-Hôpitaux de Paris, Hôpital Européen Georges Pompidou, Service d’Anatomie Pathologique, Paris, France; 11 Assistance Publique-Hôpitaux de Paris, Hôpital Européen Georges Pompidou, Service de Cardiologie, Paris, France; 12 Research Unit of Molecular Epidemiology, Helmholtz Zentrum München, German Research Center for Environmental Health, Neuherberg, Germany; 13 Institute of Epidemiology, Helmholtz Zentrum München, German Research Center for Environmental Health, Neuherberg, Germany; 14 German Center for Diabetes Research (DZD), Neuherberg, Germany; 15 German Research Center for Cardiovascular Research (DZHK), Partner Site Munich Heart Alliance, Munich, Germany; 16 Institute of Biomedical Technologies National Research Council of Italy, Milan, Italy; 17 Bio4Dreams-Business Nursery for Life Sciences, Milan, Italy; 18 Neuroalgology Unit, Fondazione IRCCS Istituto Neurologico ‘Carlo Besta’, Milan, Italy; 19 UMR_1142, INSERM, Sorbonne Université, Université Paris 13, Paris, France; 20 Sorbonne Paris Nord University, INSERM U1153, INRAe U1125, CNAM, Nutritional Epidemiology Research Team (EREN), Epidemiology and Statistics Research Center – Université Paris Cité (CRESS), 93017 Bobigny, France; 21 Assistance Publique-Hôpitaux de Paris, Hôpital Européen Georges Pompidou, Service de Génétique, Paris, France

**Keywords:** Adrenal gland diseases, Hypertension, Genome-wide association studies

## Abstract

Primary aldosteronism affects up to 10% of hypertensive patients and is responsible for treatment resistance and increased cardiovascular risk. Here we perform a genome-wide association study in a discovery cohort of 562 cases and 950 controls and identify three main loci on chromosomes 1, 13 and X; associations on chromosome 1 and 13 are replicated in a second cohort and confirmed by a meta-analysis involving 1162 cases and 3296 controls. The association on chromosome 13 is specific to men and stronger in bilateral adrenal hyperplasia than aldosterone producing adenoma. Candidate genes located within the two loci, *CASZ1* and *RXFP2*, are expressed in human and mouse adrenals in different cell clusters. Their overexpression in adrenocortical cells suppresses mineralocorticoid output under basal and stimulated conditions, without affecting cortisol biosynthesis. Our study identifies the first risk loci for primary aldosteronism and highlights new mechanisms for the development of aldosterone excess.

## Abstract (web summary)

Detection of primary aldosteronism, the most common form of secondary arterial hypertension, is essential for targeted management and prevention of cardiovascular complications. Here, the authors identify genetic loci associated with primary aldosteronism, suggesting new mechanisms of disease.

## Introduction

High blood pressure is the leading global contributor to premature death, accounting for more than 10 million deaths and over 200 million disability-adjusted life years^1,2^. Remarkably, each increment of 20 mmHg in SBP is associated with about a twofold difference in age-specific mortality rates from stroke, ischemic heart disease, and other vascular causes^3^. Despite this ample evidence, in two thirds of patients, blood pressure is not controlled sufficiently to reach accepted targets^4^. Detection of secondary forms of hypertension is key to targeted management and prevention of cardiovascular complications. PA is the most common form of secondary and curable hypertension, with a prevalence of 5% in primary care and up to 10% in referred patients^5,6^; its prevalence increases with severity of hypertension and reaches up to 20% in patients with resistant hypertension^7^. PA is due to autonomous and excessive aldosterone production from the adrenal cortex, due to an aldosterone producing adenoma (APA), which can be treated by unilateral adrenalectomy, or bilateral adrenal hyperplasia (BAH)^8^. Patients are diagnosed based on a clinical case finding with hypertension and an increased aldosterone to renin ratio (ARR), often associated with hypokalemia. PA is associated with a risk of cardiovascular complications, in particular stroke, coronary artery disease, atrial fibrillation, and heart failure that exceeds that of patients with essential hypertension^9^. Due to the complexity of the work-up, the diagnosis of PA is frequently overlooked and consequently treatment of the condition is either not initiated or delayed by several years after hypertension onset, when cardiovascular complications are established.

There is growing evidence for inappropriate aldosterone production playing a role in a larger subset of patients with hypertension. Plasma aldosterone and renin levels and the ARR correlate with increased blood pressure and increased incidence of hypertension over time in the general population^10–12^. Recent data show that 24h-urinary aldosterone levels following an oral sodium suppression test were continuously increased throughout blood pressure categories, suggesting a continuum of renin-independent aldosterone production (and eventually undetected PA) in patients with hypertension, which parallels the severity of hypertension and may play a role in the development of high blood pressure in the general population^13^.

Mutations in genes coding for ion channels (*KCNJ5*^14^, *CACNA1D*^15,16^, *CACNA1H*^17,18^, *CLCN2*^19,20^), and ATPases (*ATP1A1* and *ATP2B3*,^15,21^), have been identified in a majority of APA^22,23^ and in Mendelian forms of PA^24^. All these genes regulate intracellular ion homeostasis and/or plasma membrane potential, and mutations lead to increased intracellular calcium signaling, which is the main pathway regulating aldosterone biosynthesis^25^. Additional genes have been involved in APA presenting in puberty, pregnancy or menopause^26^. However, the causes underlying a large proportion of cases of PA are still unknown and the existence of common mechanisms involved in the development of APA and BAH has been evoked^27^.

Here, we hypothesize that subtle genetic variation may predispose to the development of PA. To identify genomic loci that may confer an increased susceptibility of developing PA, we conducted a genome-wide association study (GWAS) in a discovery set of 562 patients with PA and 950 controls. We identify three loci on chromosomes 1, 13, and X at a genome-wide significance (*P*  <  5 × 10^−8^), as well as a fourth locus on chromosome 11 at suggestive significance (*P* < 10^−6^); associations on chromosome 1, 11, and 13 are replicated in a second cohort and confirmed by a global meta-analysis involving 1162 cases and 3296 controls. The association on chromosome 13 is specific to men and stronger in BAH than in APA in subtype analyses. Candidate genes located within the two main loci, *CASZ1* and *RXFP2*, are expressed in human and mouse adrenals in different cell clusters and their overexpression in adrenocortical cells significantly modifies mineralocorticoid output under basal and stimulated conditions.

## Results

### GWAS reveals major loci for PA

**Fig. 1:**
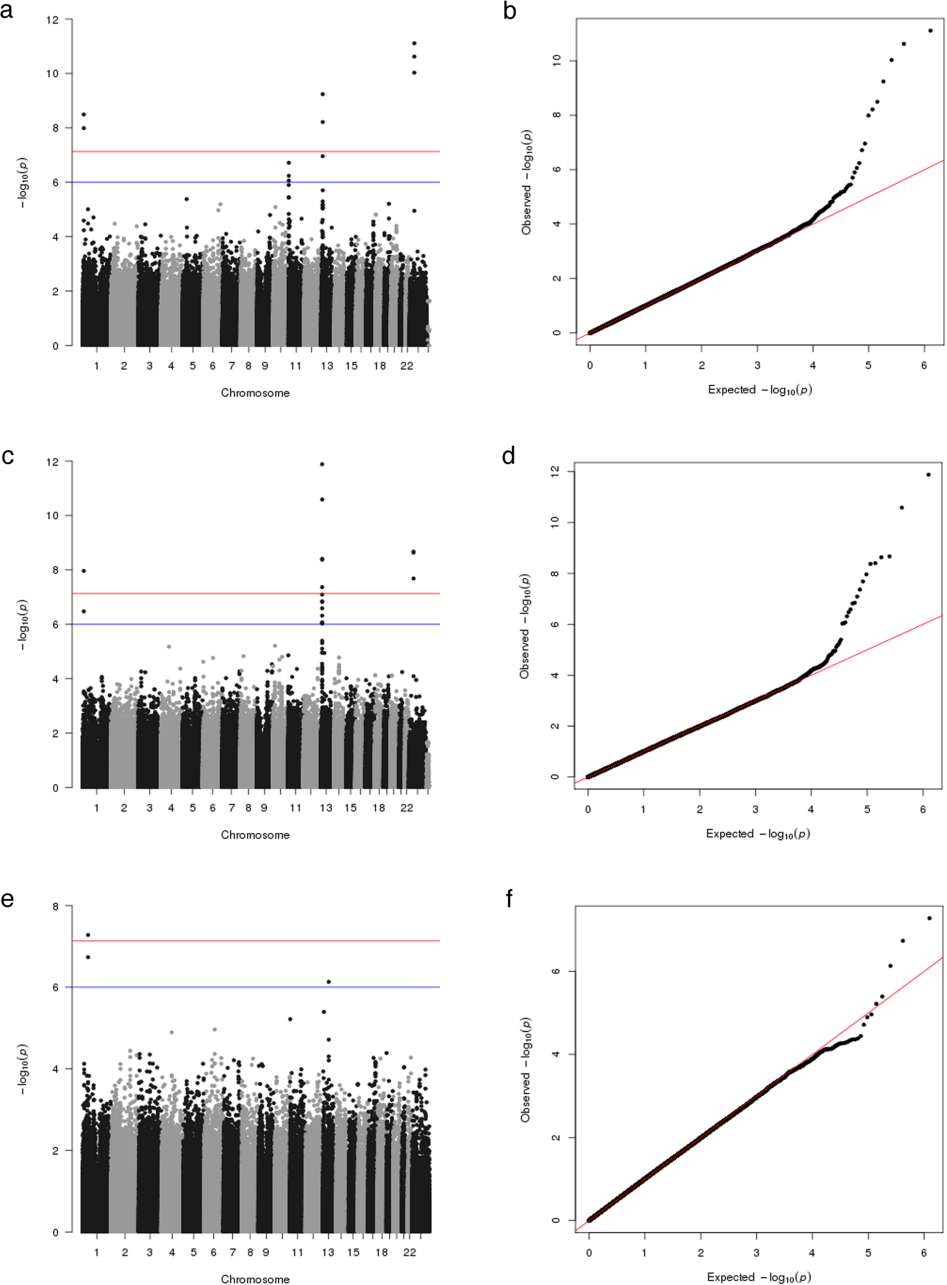
Results of the discovery-stage genome-wide association study. Manhattan plots (**a**, **c**, **e**) and quantile-quantile (QQ) plots (**b**, **d**, **f**) showing results for the entire discovery cohort (**a**, **b**) and subanalyses for men (**c**, **d**) and women (**e**, **f**). For the entire discovery cohort, three loci with genome-wide significance (*P* < 7.36 × 10^−8^) on chromosome 1, 13, and X and a fourth locus on chromosome 11 with suggestive evidence (*p* value < 10^−6^) of association were identified (**a**). For men, the three loci on chromosome 1, 13, and X reached genome-wide significance (**b**). For women, a fifth locus on chromosome 1 around 48.52 Mb was identified at genome-wide significance and a sixth locus with suggestive evidence of association on chromosome 13 around 70.1 Mb (**c**).

We conducted an initial discovery GWAS on a French dataset. We analyzed 562 PA cases (223 women and 339 men) from the Hôpital Européen Georges Pompidou (HEGP) and 950 controls from the Paris Prospective Study III (PPS3) (311 women and 639 men) for ~680,000 genotyped Single Nucleotide Polymorphisms (SNPs) (Supplementary Table 1). After a Bonferroni correction for multiple testing, three loci showed a genome-wide significant association (*P* < 7.36 × 10^−8^) (Fig. 1, Table 1 and Supplementary Data 1). The strongest association was observed on chromosome X around 43.85 Mb (lead SNP rs5905587: odds ratio (OR) = 1.596; *P* = 7.79 × 10^−12^). The second genome-wide significant locus was on chromosome 13 around 32.11 Mb (lead SNP rs1535532: OR = 1.658; *P* = 5.76 × 10^−10^). Finally, the third locus was identified on chromosome 1 around 10.79 Mb (lead SNP rs284277: OR = 1.618; *P* = 3.22 × 10^−9^). We then investigated SNPs with suggestive evidence of association with a *p* value < 10^−6^ and identified a fourth locus on chromosome 11 around 1.88 Mb (lead SNP rs2137320: OR = 1.514; *P* = 1.92 × 10^−7^) (Fig. 1 and Supplementary Data 1).

**Table 1:** Genome-wide significant associations of SNPs with PA in the discovery cohort

						All	Men		Women		APA		BAH	
SNP	chr	position	A1/A2	Freq	OR (CI)	*P* value	OR (CI)	*P* value	OR (CI)	*P* value	OR (CI)	*P* value	OR (CI)	*P* value
rs284277	1	10790797	C/A	0.38	1.62 (1.38–1.9)	3.22E-09**	1.8 (1.47–2.21)	1.09E-08**	1.35 (1.04–1.76)	0.0252	1.6 (1.33–1.94)	1.22E-06	1.61 (1.3–1.99)	1.28E-05
rs6679531	1	48524180	C/T	0.36	1.41 (1.2–1.65)	3.28E-05	1.1 (0.9–1.35)	0.350	2.14 (1.63–2.81)	5.25E-08**	1.41 (1.16–1.71)	0.000613	1.41 (1.14–1.75)	0.00188
rs2137320	11	1884342	A/G	0.42	1.51 (1.29–1.77)	1.92E-07*	1.36 (1.12–1.66)	0.00237	1.82 (1.41–2.37)	6.04E-06	1.74 (1.44–2.11)	1.18E-08**	1.3 (1.05–1.61)	0.0159
rs1535532	13	32114398	T/C	0.63	1.66 (1.41–1.95)	5.76E-10**	2.15 (1.74–2.66)	1.31E-12**	1.13 (0.87–1.46)	0.360	1.46 (1.21–1.77)	0.000107	1.98 (1.58–2.49)	3.88E-09**
rs5905587	X	43833996	C/T	0.64	1.6 (1.4–1.82)	7.79E-12**	1.59 (1.37–1.86)	2.31E-09**	1.67 (1.26–2.21)	0.000382	1.56 (1.32–1.84)	1.89E-07*	1.66 (1.38–1.99)	8.69E-08*

Lead variants with a significant or a suggestive association with the phenotype in the full discovery cohort and/or in at least one stratified analysis are presented. Associations were tested using a logistic regression model in PLINK 1.9. **significant association after Bonferroni correction (*P* < 7.36 × 10^−8^) indicated in bold, *suggestive association (*P* < 10^−6^); position: genome build 37.

*A1* risk allele, *A2* second allele, *Freq* risk allele frequency, *OR (CI)* odds ratio (95% confidence interval).

The same analyses were stratified for sex (Fig. 1 and Table 1). In men, the three loci on chromosome 1, 13, and X were found to be genome-wide significant, especially the locus on chromosome 13 (lead SNP rs1535532: OR = 2.152; *P* = 1.31 × 10^−12^) (Fig. 1c, d and Table 1), while no suggestive association was found for the fourth locus on chromosome 11. In women, none of the four loci were significantly associated with PA; however, a fifth locus was identified on chromosome 1 around 48.52 Mb that was genome-wide significant (rs6679531: OR = 2.139; *P* = 5.25 × 10^−8^), as well as a sixth locus with suggestive evidence of association on chromosome 13 around 70.1 Mb (rs569016: OR = 2.208; *P* = 7.38 × 10^−7^) (Fig. 1e, f and Supplementary Data 1).

We further performed a GWAS for each subtype of PA, APA and BAH (Supplementary Fig. 1 and Table 1). One individual not assigned to a subtype was removed. We first compared the 321 APA cases (148 women and 173 men) to the 950 PPS3 controls and found a genome-wide significant association for the locus on chromosome 11 (lead SNP rs2137320: 1.741; *P* = 1.18 × 10^−8^) that had shown suggestive evidence of association with all PA cases. Moreover, the loci on chromosomes 1 and X that showed a significant association with PA in the entire discovery cohort, showed also evidence of association with APA (Supplementary Data 1). Similarly, we compared the 240 BAH cases (74 women and 166 men) to the 950 PPS3 controls and found genome-wide significant associations for two of the three loci significantly associated with all PA cases, namely the locus on chromosome 13 (lead SNP rs1535532: OR = 1.981; *P* = 3.88 × 10^−9^, Table 1) and the locus on chromosome X (rs1005002: OR = 1.648; *P* = 5.99 × 10^−8^, Supplementary Data 1).

In total, 25 variants that are distributed over six loci were identified with an association at *P* < 10^−6^ in the discovery GWAS, in the analysis of the full discovery dataset and/or in one stratified analysis (men, women, APA or BAH) (Table 1 and Supplementary Data 1). Five of these loci (chromosome 1, lead SNP rs284277; chromosome 1, lead SNP rs6679531; chromosome 11, lead SNP rs2137320; chromosome 13, lead SNP rs1535532; and chromosome X, lead SNP rs5905587) showed a genome-wide significant association in the analysis of the full discovery dataset and/or in one stratified analysis.

To perform high-resolution mapping of association signals, we performed imputation on the discovery cohort. All previous significant and suggestive association signals were confirmed, but no additional significantly associated locus was observed at a genome-wide significance level of 5 × 10^−8^ (Supplementary Data 2 and Supplementary Fig. 2). Conditional analysis on the six loci did not reveal any significantly independent signal in the discovery cohort or in a stratified analysis, except for the locus on chromosome 11 where two independent suggestive associations for the entire cohort were identified (Supplementary Data 2 and Supplementary Fig. 2).

Heterogeneity analyses to assess the association differences observed between men and women revealed that the locus on chromosome 13 (lead SNP rs1535532) associated with PA in men only showed a significant heterogeneous association between men and women (*P* = 1.61 × 10^−4^). Heterogeneity was also observed for the two loci associated with PA in women only on chromosomes 1 (lead SNP rs6679531: *P* = 1.5 × 10^−4^) and 13 (lead SNP rs569016: *P* = 1.73 × 10^−5^) (Supplementary Table 2). In contrast, heterogeneity analyses did not confirm differences between APA and BAH in the discovery cohort (Supplementary Table 3).

Among the genotyped and imputed variants identified, 16 were available after genotyping in the German dataset and were tested for association to replicate suggestive loci. We analyzed 399 PA cases (255 men and 144 women) and 1847 KORA controls (916 men and 931 women). Three of the four significant/suggestive loci identified in the discovery cohort with all PA cases (on chromosomes 1, 11, and 13) were confirmed with a significant association in the German dataset, after Bonferroni correction for the 16 variants tested (*P* < 0.0031). In contrast, the fourth locus on chromosome X with a significant association in the discovery cohort did not show a significant association in the German dataset (lead SNP rs1005002: OR = 1.178; *P* = 0.0214) (Table 2 and Supplementary Data 3). For men, two of the three significant loci in the discovery cohort were significantly associated with PA: the locus on chromosomes 1 (lead SNP rs284277) and especially the locus on chromosome 13 (lead SNP rs1535532: OR = 2.05; *P* = 3.36 × 10^−9^), while the association of the locus on chromosome X was not replicated (Supplementary Table 4). The significant association on chromosome 1 and the suggestive one on chromosome 13 that were specific to women in the discovery cohort (lead SNPs rs6679531 and rs1535532 respectively) were not replicated among German women. In contrast, the locus on chromosome 1 (lead SNP rs284277) that had shown a significant association in the whole discovery cohort and in men, but not in women, was significantly associated with PA in German women (Supplementary Table 5). Heterogeneity analysis confirmed that the locus associated with PA in men on chromosome 13 (with lead SNP rs1535532) showed a very significantly heterogeneous association in the German cohort between men and women (*P* = 3.48 × 10^−7^) (Supplementary Table 2).

**Table 2:** Replication of genome-wide significant/suggestive associations of SNPs with PA and meta-analysis

					Discovery cohort		German cohort		German + Italian + French#2 cohorts			Global meta-analysis		
SNP	chr	position	A1/A2	#coh	OR (95% CI)	*P* value	OR (95% CI)	*P* value	OR (FE) (95% CI)	*P* value (FE)	*P* value (RE)	OR (FE) (95% CI)	*P* value (FE)	*P* value (RE)
rs284277	1	10790797	C/A	3	1.62 (1.38–1.9)	3.21E-09**	1.42 (1.2–1.67)	3.96E-05**	1.34 (1.15–1.56)	1.6E-04**	0.000193**	1.47 (1.31–1.64)	9.12E-12**	3.60E-11**
rs6679531	1	48524180	C/T	3	1.41 (1.2–1.65)	3.28E-05	1.14 (0.96–1.35)	0.133	1.16 (0.99–1.35)	0.0623	0.0716	1.27 (1.14–1.42)	2.48E-05	4.24E-05
rs2137320	11	1884342	A/G	4	1.51 (1.29–1.77)	1.92E-07*	1.31 (1.1–1.55)	0.00208**	1.38 (1.2–1.59)	7.08E-06**	9.59e-06**	1.44 (1.3–1.6)	8.78E-12**	3.57E-11**
rs1535532	13	32114398	T/C	4	1.66 (1.41–1.95)	5.76E-10**	1.41 (1.18–1.67)	0.000115**	1.28 (1.1–1.47)	8.95E-04**	0.00103**	1.43 (1.29–1.59)	3.95E-11**	2.12E-11**
rs5905587	X	43833996	C/T	3	1.6 (1.4–1.82)	7.79E-12**	1.13 (0.99–1.31)	0.0785	1.15 (1–1.3)	0.0436	0.0503	1.35 (1.23–1.48)	4.75E-10**	4.56E-12**

Lead variants with a significant or a suggestive association with the phenotype in the full discovery cohort and/or in at least one stratified analysis.

Associations were tested using a logistic regression model in PLINK 1.9. The replication meta-analysis and the joint analysis of the discovery and replication stages were carried out as a fixed effect inverse-variance or a random-effects model meta-analysis using METASOFT.

*A1* risk allele in the full discovery cohort, *A2* second allele, *#coh* number of cohorts where the SNP is available, *OR (95% CI)* odds ratio (95% confidence interval), *FE* fixed-effect model, *RE* random-effect model.

*Suggestive association for genotyped data in the discovery cohort (*P* < 10^−6^).

**Significant association after Bonferroni correction (*P* < 7.36 × 10^−8^ for genotyped data and *P* < 5 × 10^−8^ for imputed data in the discovery cohort; *P* < 7.36 × 10^−8^ for the global meta-analysis; *P* < 0.0031 for the German replication cohort and for the replication meta-analysis of the German, Italian and second French cohorts).

We then compared the 214 German APA cases (135 men and 79 women) to 1847 KORA controls and found two significant associations for the locus on chromosome 1 that had shown a suggestive association with APA in the discovery cohort (lead SNP rs284277) and for the locus on chromosome 13 that had shown a significant association with all PA cases in the discovery cohort (lead SNP rs1535532) (Supplementary Table 6). Moreover, the locus on chromosome 11 identified with a significant association with APA in the discovery cohort showed an association with APA close to the replication significance level in the German dataset (lead SNP rs2137320: OR = 1.356; *P* = 0.00615), while the suggestive association on chromosome X with APA in the discovery cohort was not replicated. Similarly, we compared the 135 German BAH cases (91 men and 44 women) and 1847 KORA controls and replicated the significant association with the locus on chromosome 13 (lead SNP rs1535532), while the other significant association on chromosome X in the discovery cohort was not replicated (Supplementary Table 7).

Very similar results were obtained by a meta-analysis combining this German replication dataset and two smaller replication datasets (an Italian dataset, with 107 patients (76 APA, 31 BAH) and 300 normotensive population controls and a second French dataset, with 94 patients (64 APA, 30 BAH) and 199 SUVIMAX controls) (Supplementary Tables 4–9 and Supplementary Data 3). Interestingly, the significant association of the locus on chromosome 11 (lead SNP rs2137320) with APA was replicated in this meta-analysis and a significant replication signal was also observed with PA in men and in women for this locus, while in the German cohort the association was only replicated with the entire dataset.

In conclusion, the significant/suggestive associations of three loci (chromosome 1, lead SNP rs284277; chromosome 11, lead SNP rs2137320; and chromosome 13, lead SNP rs1535532) in the analysis of the full discovery dataset and/or in one stratified analysis were replicated in the German dataset and in the meta-analysis performed on the three replication cohorts, while the associations of the locus on chromosome X and of the two women-specific loci in the discovery cohort were not.

Finally, in order to combine the results obtained in the discovery and replication datasets, we performed a global meta-analysis, using the four cohorts (Table 2 and Supplementary Tables 10 and 11). Among the 26 variants showing an association at *P* < 10^−6^ in our discovery GWAS, 18 variants were available in at least another dataset and thus considered in the global meta-analysis. Almost all the genome-wide significant associations observed in the discovery GWAS (either in the analysis of the full discovery dataset or in one stratified analysis) were confirmed by the global meta-analysis (*P*  < 7.36 × 10^−8^), both with the fixed-effect and the random-effect models (Table 2 and Supplementary Tables 10 and 11). The two exceptions were the association of the locus on chromosome X, which only reached a suggestive significance level for BAH in the global meta-analysis (lead SNP rs1005002; OR = 1.432; *P* = 1.91 × 10^−7^ with the random-effect model) but remained significant for all PA and in men, and the association observed in women on chromosome 1 (lead SNP rs6679531), which was already the least significant in the discovery cohort and did not even reach suggestive significance in the fixed-effect global meta-analysis model. Four of the five loci with a significant association in the full discovery dataset and/or in one stratified analysis (the three loci whose association was replicated in the replication datasets and the locus on chromosome X) were thus significantly associated in the global meta-analysis as well. The sixth locus located on chromosome 13, which showed suggestive evidence of association in the discovery cohort in the analysis restricted to women, also showed a suggestive association in the random-effect model (lead SNP: rs569016; OR = 1.356; *P* = 3.61 × 10^−7^). Finally, heterogeneity analysis confirmed that the locus associated with PA in men on chromosome 13 (lead SNP rs1535532) showed a very significantly heterogeneous association between men and women (*P* = 5.68 × 10^−11^) (Supplementary Table 2); this locus also showed a significant heterogeneity between APA and BAH in the global meta-analysis (lead SNP rs1535532: *P* = 3.39 × 10^−3^) (Supplementary Table 3).

### Identification of potential genes associated with PA

**Fig. 2:**
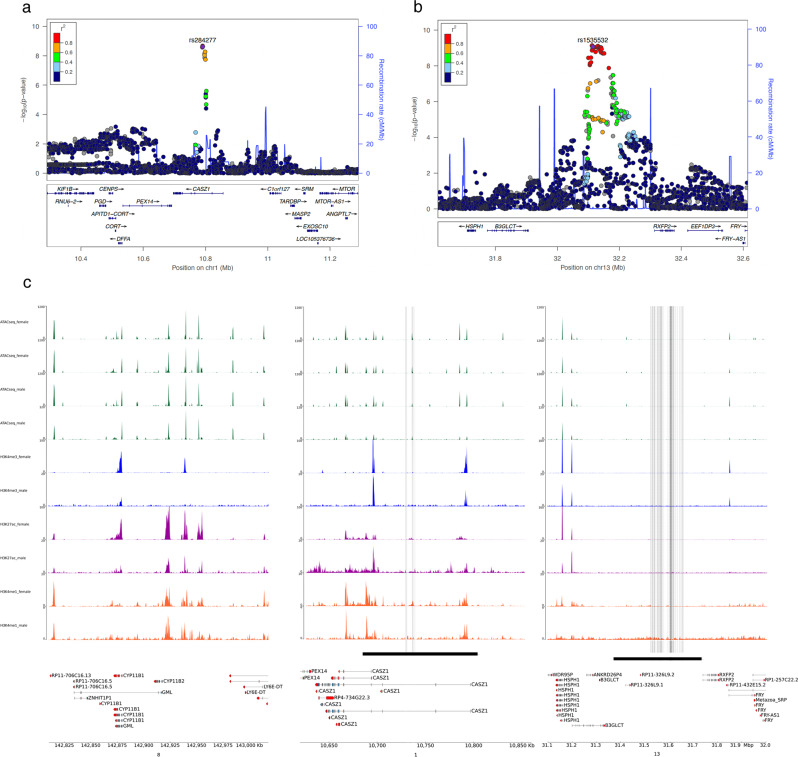
Genomic context of the association signals observed in the GWAS in the discovery cohort. The regional association plots were generated using LocusZoom on imputed data and display surrounding genes (genome build 37). The two different loci on chromosome 1 (**a**) and chromosome 13 (**b**) confirmed in the meta-analysis are represented; dot color indicates linkage disequilibrium of each variant with the highlighted lead variant in common between discovery and replication cohorts (purple diamond). **c** Genome browser view of public ATAC-seq and ChIP-seq signals produced by the ENCODE consortium from adrenal gland tissue on chromosome 8 (region 142,810,559–143,017,843, hg38 build), chromosome 1 (region 10,620,000–10,860,000) and chromosome 13 (region 31,100,000−32,000,000). Top tracks (in green) are ATAC-seq signals from four donors (two females and two males). The lower tracks are ChIP-seq results from three different donors (one female and two males). For each sex, we show tracks corresponding to three histone marks, known to be associated with regulatory elements: H3K4me3 (blue), H3K27ac (purple), and H3K4me1 (orange). The black horizontal bar delimitates the regions in linkage disequilibrium, found associated with PA. Vertical dashed lines indicate the position of SNPs identified in this study.

Within the four main loci associated with PA at genome-wide significance level in the full discovery dataset and/or in one stratified analysis, a total of 51 protein coding genes are located within a 1 Mb interval around the identified variants (Fig. 2, and Supplementary Fig. 3). The lead SNP rs284277 on chromosome 1, as well as the other SNPs of the locus, cluster within the *CASZ1* gene, while the locus on chromosome 13 (top SNP rs1535532) lies upstream of *RXFP2* (Fig. 2a, b). On the X-chromosome, (top SNP rs5905587) SNPs are located within or in proximity to the *NDP* gene (Supplementary Figure 3a). On chromosome 11, rs2137320 is located within the *LSP1* gene (Supplementary Fig. 3c). eQTL analysis using the Genotype-Tissue Expression (GTEx) project showed that all SNPs on the locus on chromosome 13 are top eQTLs for *RXFP2* in the adrenal gland and most variants on the X chromosome are eQTLs for *NDP* (Supplementary Table 12, Supplementary Data 4 and www.gtexportal.org). In addition, rs2137320 and rs509239 on chromosome 11 are sQTLs for LSP1, possibly modifying splicing patterns. Remarkably, all risk alleles on chromosome 13 are associated with an increased expression of RXFP2 in the adrenal gland (Supplementary Table 12 and Supplementary Data 4).

The other two loci identified exclusively in women in the discovery cohort are on chromosome 1 and 13. Four protein-coding genes are located within a 1 Mb interval on chromosome 1 around rs6679531 and two genes are located within 1 Mb from rs 569016 on chromosome 13 (Supplementary Fig. 3d, e). For none of those the identified SNPs are eQTL in GTEX, nor are those SNPs eQTLs for any other gene in the adrenal gland.

The SNPs at *CASZ1* and SNPs in linkage disequilibrium with SNPs at the *RXFP2* locus associate with different blood pressure related traits^28,29^ (https://www.ebi.ac.uk/gwas). Similarly, several SNPs in linkage disequilibrium with the top SNP rs2137320 on chromosome 11 and a SNP in linkage disequilibrium with rs1005002 and rs5905587 on chromosome X have been associated with blood pressure^30,31^. In addition, SNPs in *CASZ1* have been associated with treatment resistant hypertension in the CHARGE consortium^32^. Recently, SNPs near *CASZ1*, *LSP1* and *RXFP2* have been associated with resistant hypertension in a genome-wide association study of 14,756 patients from Iceland, the UK Biobank and eMERGE^33^. The risk alleles were associated with lower potassium levels and the effect on potassium predicted their association with resistant hypertension beyond their blood pressure effect. These results suggested an implication for aldosterone and the mineralocorticoid pathway in the pathogenesis of resistant hypertension. In this context it is noteworthy that the prevalence of PA increases to up to 20% in patients with resistant hypertension^34–36^ and that it was the clinical presentation in 20–50% of the patients with PA submitted to adrenal vein sampling in the AVIS-2 Study^37^.

### Chromatin regulatory landscape of loci associated with PA and expression in adrenal gland

Given that the main loci associated with genome-wide significance with PA in the discovery cohort and replicated in the large German cohort are located on chromosome 1 and 13, we focused on those candidates for subsequent studies. The protein encoded by *CASZ1* is a zinc finger transcription factor expressed in multiple tissues. Inactivation of CASZ1 in mice leads to abnormal heart development^38^ and CASZ1 loss-of-function mutations associate with congenital heart disease^39^. In addition, CASZ1 may also function as a tumor suppressor^40^. Genes regulated by CASZ1 include *CACNA1D* and *KCNK3*^38^, two channel genes known to be involved in human and mouse PA. In particular, gain of function mutations in *CACNA1D* are found in APA and a rare syndromic form of PA^16^ and inactivation of *KCNK3* in mice leads to a phenotype of glucocorticoid suppressible aldosteronism^41^. In addition, it has recently been shown that CASZ1 is a corepressor of the transcriptional activity of the mineralocorticoid receptor (MR)^42^, which mediates aldosterone effects in target tissues and is also specifically expressed in the adrenal zona glomerulosa^43^. Remarkably, *CASZ1* has recently been identified as a differentially expressed gene between aldosterone producing cell clusters (APCC) and zona glomerulosa cells in the adrenal cortex^44^. APCC, also called aldosterone producing micronodules^45^, are clusters of cells located in the zona glomerulosa, which express aldosterone synthase^43,46^; APCC carry somatic mutations and are considered possible precursors of APA^44^. *RXFP2* encodes a G protein-coupled, 7-transmembrane receptor for the relaxin family peptide insulin-like peptide 3 (INSL3) that signals through Gαs to increase cAMP^47^, which acts as second messenger for several aldosterone secretagogues^48^. RXFP2 controls testicular descent in mice and RXFP2 mutations have been associated with familial cryptorchidism^49^. Knockdown of RXFP2 reduced *CYP17A1* expression and androstenedione production in cultured bovine theca cells^50^, suggesting a possible role in steroid hormone biosynthesis. Publicly available RNA sequencing data (GTEx, Human Protein Atlas) show that, albeit being expressed mainly in the testis and epididymis and at lower levels in ovary, RXFP2 is also expressed in the adrenal gland. Expression of *CASZ1* and *RXFP2* in the adrenal cortex was retrieved from a transcriptome study including 11 human control adrenals and 123 APA^51^ (Supplementary Fig. 4). CASZ1 mRNA was expressed in control adrenals and showed a significant upregulation in APA (Supplementary Fig. 4a). Differences in *CASZ1* mRNA expression were also observed by RT-qPCR on a smaller set of samples, although this did not reach statistical significance (Supplementary Fig. 4c). *RXFP2* was expressed similarly in both control adrenals and APA (Supplementary Fig. 4b and d). Overall, expression of *CASZ1* and *RXFP2* was heterogeneous in APA and no differences were observed between APA carrying different somatic mutations (*n* = 122, RXFP2, *p* = 0.9167; CASZ1, *p* = 0.2113 Kruskal-Wallis test).

To explore the chromatin regulatory landscape in the adrenal gland in the two PA-associated loci located on chromosome 1 and 13, we investigated datasets from the ENCODE project. We profiled regions of open chromatin using ATAC-seq signals^52^ and analyzed the distribution of three histone marks associated with regulatory elements: H3K4me3, H3K27ac and H3K4me1^53,54^ (Fig. 2c). Several regions of accessible chromatin were observed within *CASZ1* on chromosome1, with a common pattern between males and females. Two open chromatin regions mapped at the level of the two *CASZ1* alternative transcription start sites. These regions are also positive for H3K4me3 and H3K27ac, two histone marks typically associated at active promoters. One additional open chromatin region was also detected outside of the promoter regions, inside a *CASZ1* intron. The region was enriched in H3K27ac and H3K4me1, which suggests that it corresponds to an active enhancer element. The two *CASZ1* promoters and the putative enhancer are all located within the region in linkage disequilibrium. Their function could be altered by genetic variations that affect *CASZ1* expression and thus participate to the disease. In contrast, we could not detect open chromatin regions in chromosome 13 within the PA-associated locus that contains the *RXFP2* gene. To verify the hypothesis that the absence of ATAC-seq signal within this locus could be due to an insufficient representation in the ENCODE datasets of cells expressing *RFXP2*, we analyzed the epigenetic landscape of two control genes, *CYP11B1* and *CYP11B2*. *CYP11B1* is expressed throughout the zona fasciculata, the largest part of the adrenal cortex, while *CYP11B2* is expressed only in the zona glomerulosa, which represents only a few layer of cells underneath the capsule. Using the ENCODE dataset, we were able to detect H3K4me3 at the *CYP11B1* promoter, but not at *CYP11B2*. This observation strongly suggests that the investigated datasets on the whole adrenal gland do not provide sufficient information to detect signals for genes expressed in a restricted subset of cells.

**Fig. 3:**
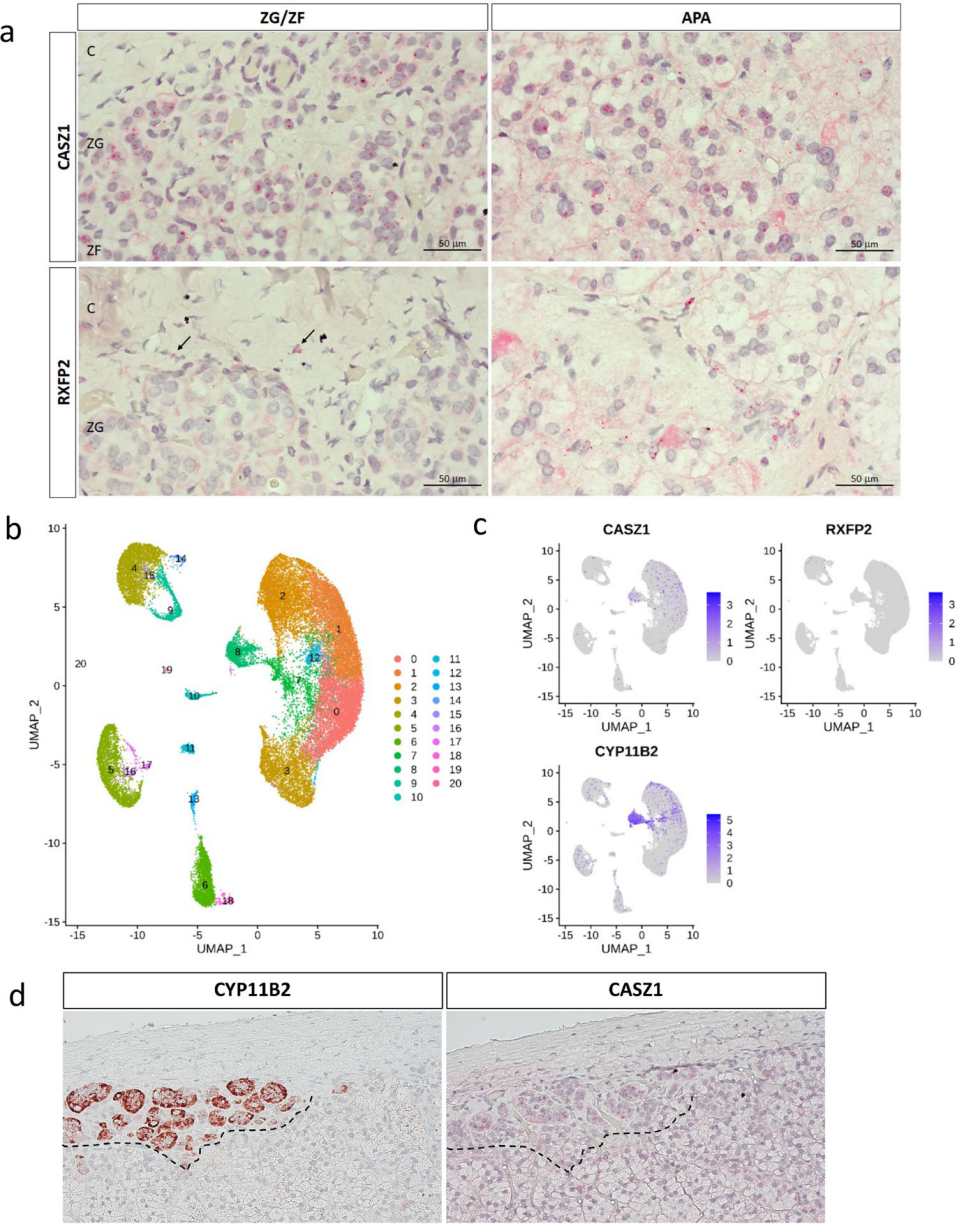
Expression of *CASZ1* and *RXFP2* in the adrenal cortex and APA. **a** *CASZ1* and *RXFP2* mRNA localization in the adrenal cortex of an adrenal with APA carrying a somatic *KCNJ5* mutation analyzed by RNAscope. Red dots represent positive staining. Images are representative of results obtained in adrenals from four patients. C capsule, ZG zona glomerulosa, ZF zona fasciculata. **b** Uniform Manifold Approximation and Projection (UMAP) diagram from snRNA-seq from 5 pieces of adrenal cortex adjacent to APA from two adrenals; cells are colored by 1–20 cell-type clusters. **c** UMAP plots showing the expression of *CASZ1*, *RXFP2*, and *CYPP11B2* within the different clusters. **d** *CASZ1* mRNA localization in cells expressing CYP11B2 in the adrenal cortex of an adrenal with APA (patient 1 analyzed by snRNAseq) carrying a somatic *KCNJ5* mutation. *CASZ1* mRNA expression was analyzed by RNAscope; CYP11B2 protein expression was analyzed by immunohistochemistry. For each sample, one experiment was performed.

The expression of *CASZ1* and *RXFP2* was thus explored by RNAscope in the adrenal cortex of adrenals with APA. *CASZ1* was mainly expressed in the zona glomerulosa extending into the zona fasciculata, albeit at lower levels; staining was also observed in APA (Fig. 3a and Supplementary Fig. 5). *RXFP2* mRNA expression was localized mainly to sub-capsular cells and the outer zona glomerulosa; expression in APA was low. To identify cells expressing *CASZ1* and *RXFP2*, we interrogated single-nucleus RNA-sequencing (snRNAseq) data generated on five adjacent tissues from two adrenal glands from patients with APA. By using Seurat software25 and Uniform Manifold Approximation and Projection (UMAP) clustering we were able to detect a total of 20 different cell clusters (Fig. 3b, left panel). *CASZ1* and *RXFP2* were projected within the different clusters as well as *CYP11B2* to identify aldosterone producing cells (Fig. 3b, right panel). The origin of the clusters expressing *CASZ1* and *RXFP2* was identified using specific cell markers (Supplementary Fig. 6). *CASZ1* was co-expressed with *CYP1B2* in seven clusters, in particular in cluster 8, which expresses top genes distinguishing APCC from zona glomerulosa cells^44^. Both genes are expressed to a lesser extent in other steroidogenic clusters. In contrast, *RXFP2* was expressed mainly in clusters 4 and 9, presenting a transcriptional profile of adrenal stem/progenitor cells, expressing both stem as well as steroidogenic cell markers^55,56^. As *CASZ1* appears to be enriched in APCC, we have also specifically adressed the expression of *CASZ1* analyzed by RNAscope in aldosterone producing regions of the zona glomerulosa in one adrenal presenting small APCC by CYP11B2 immunohistochemistry, which shows that *CASZ1* is highly coexpressed with *CYP11B2* in these structures (Fig. 3c). Similarly, in mouse adrenals, *Casz1* was strongly expressed in the zona glomerulosa and outer zona fasciculata and co-localized with *Cyp11b2*. *Rxfp2* expression was confirmed in sub-capsular cells and the zona glomerulosa and co-staining with *Cyp11b2* was observed in some cells (Supplementary Fig. 7). There was no major difference between male and female mice (Supplementary Fig. 7).

We further interrogated gene expression data from a published genomic atlas on human adrenal and gonadal development^57^. Data encompassed gene expression of 53 samples from the adrenal gland (*n* = 17), control tissue (*n* = 6, spine, brain, muscle, heart, kidney, and liver), ovary (*n* = 10) and testis (*n* = 20), from 42.5 days to 73.5 days post conception, encompassing the most important period for adrenal and gonadal development (Supplementary Fig. 8). Remarkably, *RXFP2*, considered a gonadal-specific gene, showed significantly higher expression in adrenal glands compared to control tissues and similar to ovary and testis (Supplementary Fig. 8a); *RXFP2* expression significantly decreased with developmental age (Supplementary Fig. 8b). *CASZ1* expression in the adrenal cortex was not different compared with control tissues, but significantly higher than in gonads, and did not show major changes over time (Supplementary Fig. 8c and d).

### CASZ1 and RXFP2 affect adrenocortical function

For a subset of 47 patients with APA included in the GWAS, detailed genetic analysis by aldosterone synthase-guided next generation sequencing, histological information and steroid profiles were available^23^. Aldosterone synthase is responsible for the last enzymatic steps of aldosterone biosynthesis. No association was observed between genotypes at chromosomes 1 and 13 with the somatic mutation status of the APA. There was also no association between genotypes and the presence of zona glomerulosa hyperplasia, the number of secondary nodules or the number of APCC of resected adrenal glands. Genotypes at chromosome 13 near *RXFP2* showed a tendency towards being associated with steroid output in patients with APA (Supplementary Fig. 9). In particular, the risk allele at rs1535532 was associated with lower plasma aldosterone levels (*p* = 0.033, Supplementary Fig. 9c); carriers of the risk alleles at rs1869799 and rs1902272 also showed lower, but not significantly different, aldosterone levels (Supplementary Fig. 9a, e). In addition, carriers of the risk allele at rs1869799 and rs1535532 also showed a trend towards higher cortisol over aldosterone ratio (Supplementary Fig. 9b, d), and carriers of the risk allele at rs1902272 a trend towards lower corticosterone levels (Supplementary Fig. 9f).

**Fig. 4:**
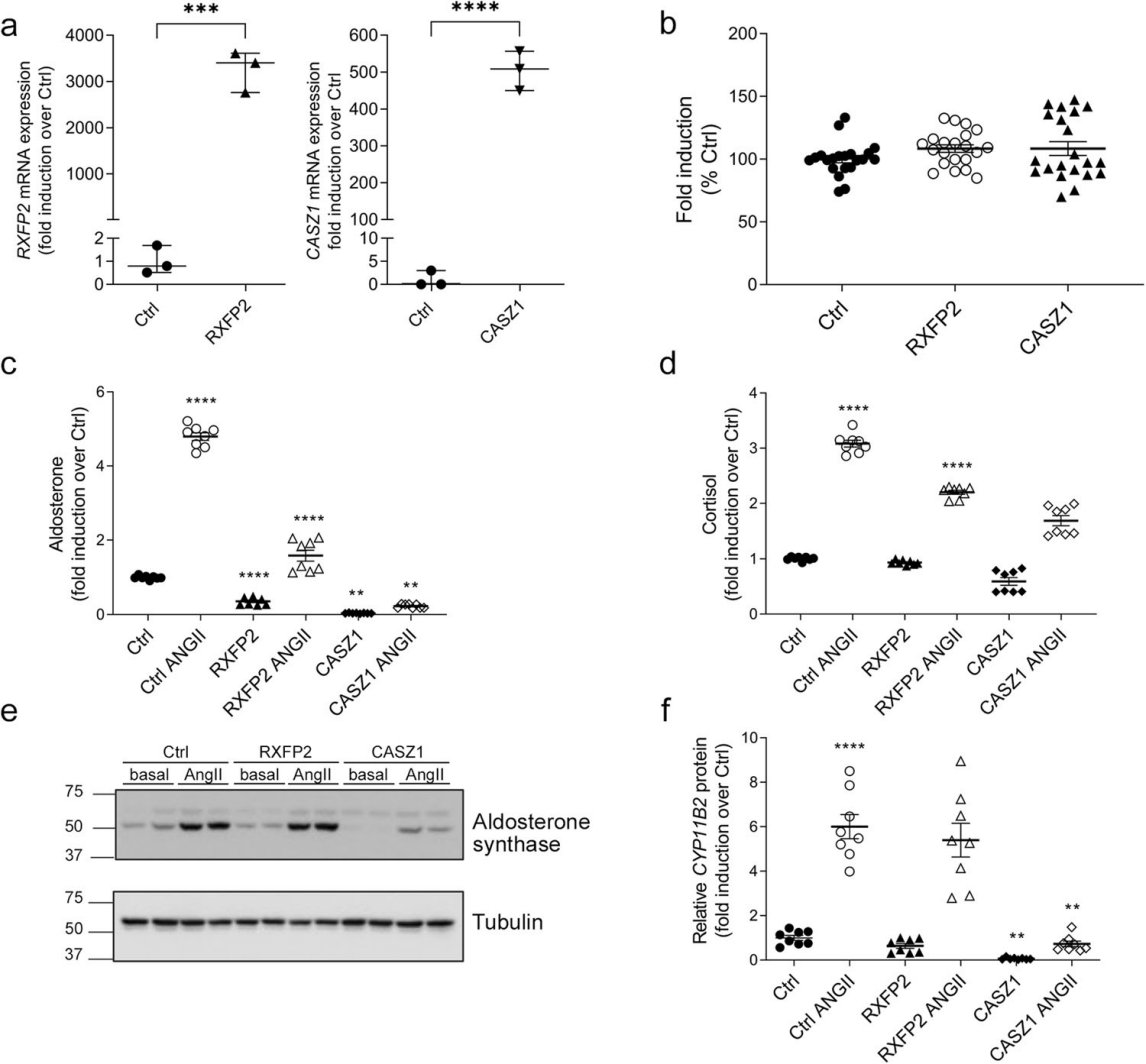
Effect of RXFP2 and CASZ1 overexpression in H295R-S2 cells. **a** RXFP2 and CASZ1 mRNA expression in H295R-S2 cells stably transfected with expression vectors coding for pcDNA3 (Ctrl), RXFP2 (RXFP2), or CASZ1 (CASZ1). mRNA expression is normalized against the geometric mean of three housekeeping genes. *RXFP2* vs Ctrl, ***p = 0.0002; *CASZ1* vs Ctrl ****p < 0.0001; unpaired *t* test, two-sided. **b** Effect of RXFP2 and CASZ1 overexpression in stably transfected H295R-S2 cells on cell proliferation. Cells were seeded in 96-well plates at a density of 5000 cells/well and grown for 4 days. Basal and stimulated aldosterone (**c**) and cortisol (**d**) production by H295R-S2 cells stably expressing RXFP2 or CASZ1. H295R-S2 cells were serum deprived for 24 h and then incubated for another 24 h with fresh serum deprived medium in absence (basal) or presence of 10 nM AngII. Aldosterone and cortisol levels are indicated as fold induction over mock-transfected cells in basal conditions. Aldosterone: Ctrl+AngII vs Ctrl ****p < 0.0001; RXFP2 vs Ctrl ****p < 0.0001; CASZ1 vs Ctrl **p = 0.0013; RXFP2 + AngII vs Ctrl+AngII ****p < 0.0001; CASZ1 + AngII vs Ctrl+AngII **p = 0.0013. Cortisol: Ctrl+AngII vs Ctrl ****p < 0.0001; RXFP2 + AngII vs Ctrl+AngII ****p < 0.0001. **e** Representative Western blot of aldosterone synthase expression in H295R-S2 cells stably expressing RXFP2 and CASZ1. **f** Quantification of aldosterone synthase expression (using tubulin as a loading control). Values are represented as mean + SEM of two independent experiments performed in quaduplicate (in **a**, median + interquartile range of one experiment performed in triplicate). Ctrl+AngII vs Ctrl ****p < 0.0001; CASZ1 vs Ctrl **p = 0.0052; CASZ1 + AngII vs Ctrl+AngII **p = 0.0052. **c**, **d**, **f**: Global comparison was evaluated using ANOVA or Kruskall Wallis test followed by Sidak’s or Dunn’s multiple comparison test. Cells overexpressing RXFP2 or CASZ1 were analyzed separately. Comparison between Ctrl vs Ctrl+Ang was performed with two-sided unpaired *t* test to control for cell response to AngII.

The role of CASZ1 and RXFP2 on adrenocortical cell function was explored in adrenocortical H295R-S2 cells. Expression of CASZ1 and RXFP2 in H295R-S2 cells significantly affected steroid production, without modifying cell proliferation (Table 3 and Fig. 4a, b). In mock transfected cells, angiotensin II (AngII 10 nM) significantly increased biosynthesis of mineralocorticoid hormones, including 18-hydroxy-11-deoxycorticosterone, corticosterone, 18-hydroxycorticosterone and aldosterone (Table 3 and Fig. 4c). In H295R-S2 cells, AngII also stimulates cortisol production (Table 3 and Fig. 4d). In comparison to H295R-S2 mock-transfected cells, cells overexpressing RXFP2 showed significantly suppressed mineralocorticoid output (11-deoxycorticosterone, 18-hydroxy-11-deoxycorticosterone, corticosterone, aldosterone) both under basal and stimulated conditions. Basal and stimulated 21-deoxycortisol was increased, suggesting a shift in the steroidogenic pathway. Basal cortisol production was unchanged. The effects of CASZ1 overexpression in H295R-S2 cells were even more pronounced. In comparison to mock-transfected cells, CASZ1 expression completely suppressed basal and AngII induced mineralocorticoid biosynthesis (11-deoxycorticosterone, 18-hydroxy-11-deoxycorticosterone, corticosterone, 18-hydroxycorticosterone, aldosterone, Table 3 and Fig. 4c), but did not modify glucocorticoid production (Table 3 and Fig. 4d). CASZ1 expression also significantly reduced aldosterone synthase expression both in basal and stimulated conditions compared with mock-transfected cells (Fig. 4e, f), whereas no modification of aldosterone synthase expression was observed in RXFP2 overexpressing cells.

**Table 3:** Steroid profiles in stably transfected H295R_S2 cells expressing RXRP2 and CASZ1

Characteristics	Ctrl+AngII	RXFP2	RXFP2 + AngII	*p* value	CASZ1	CASZ1 + AngII	*p* value
Pregnenolone	0.90 (0.69,1.42)	0.50* (0.44,0.62)	0.36*** (0.29,0.48)	<0.0001	0.57** (0.44,0.75)	0.55** (0.34,0.65)	<0.0001
Progesterone	0.48**** (0.44,0.50)	1.23 (0.92,1.55)	0.36 (0.28,0.43)	<0.0001	1.20 (0.77,1.57)	0.53 (0.31,0.76)	<0.0001
11-deoxycorticosterone (pmol/L)	0.35**** (0.32,0.37)	0.45**** (0.37,0.53)	0.30 (0.28,0.33)	<0.0001	0.19*** (0.14,0.25)	0.17** (0.11,0.22)	<0.0001
18-hydroxy-11-deoxycorticosterone (pmol/L)	2.73*** (2.49,2.94)	0.27** (0.12,0.40)	0.41*** (0.39,0.42)	<0.0001	0.06* (0.05,0.08)	0.07**** (0.04,0.10)	<0.0001
Corticosterone (nmol/L)	4.16**** (3.64,4.71)	0.24*** (0.22,0.28)	0.97**** (0.96,1.02)	<0.0001	0.03**** (0.03,0.04)	0.19**** (0.18,0.20)	<0.0001
18-hydroxycorticosterone (pmol/L)	3.32*** (3.13,3.65)	0.51 (0.48,0.55)	1.40 (1.22,1.61)	<0.0001	0.18** (0.14,0.22)	0.42** (0.39,0.45)	<0.0001
Aldosterone (pmol/L)	4.86**** (4.53,5.00)	0.34**** (0.27,0.45)	1.57**** (1.18,2.02)	<0.0001	0.03** (0.03,0.04)	0.22** (0.18,0.28)	<0.0001
17-hydroxyprogesterone (nmol/L)	0.24**** (0.23,0.25)	1.97 (1.46,2.32)	0.35 (0.28,0.42)	<0.0001	1.56 (1.04,2.17)	0.56 (0.36,0.78)	<0.0001
11-deoxycortisol (nmol/L)	0.32*** (0.21,0.41)	1.32 (1.05,1.51)	0.72 (0.70,0.78)	<0.0001	0.78 (0.67,0.88)	0.55 (0.45,0.62)	<0.0001
Cortisol (nmol/L)	3.08**** (2.94,3.15)	0.92 (0.90,0.97)	2.24**** (2.08,2.28)	<0.0001	0.570 (0.40,0.78)	1.67 (1.44,1.94)	<0.0001
21-deoxycortisol (pmol/L)	0.70** (0.58,0.89)	2.02* (1.66,2.85)	1.49* (0.84,1.72)	0.0001	1.31 (0.38,2.66)	1.05 (0.55,1.78)	0.4796
Delta-4-androstenedione (nmol/L)	0.18**** (0.14,0.21)	0.984* (0.84,1.03)	0.22 (0.22,0.24)	<0.0001	0.69**** (0.59,0.82)	0.30** (0.23,0.37)	<0.0001

Results are expressed as fold induction over mock-transfected untreated cells (Ctrl set as 1 not shown in the table) and represent median and interquartile range, compared with ANOVA followed by Sidak’s or Kruskal-Wallis followed by Dunn’s multiple comparison test. Cells overexpressing RXFP2 or CASZ1 were analyzed separately. Comparison between Ctrl vs Ctrl+Ang was performed with unpaired t-test or Mann-Whitney test to control for response of cells to AngII.

*Ctrl* Control, *AngII* angiotensin II.

Ctrl+AngII compared to Ctrl: ****p ≤ 0.0001; 18-hydroxy-11-deoxycorticosterone *p* = 0.0002; 18-hydroxycorticosterone *p* = 0.0002; 11-deoxycortisol *p* = 0.0002; 21-deoxycortisol *p* = 0.0082.

RXFP2 vs Ctrl: ****p ≤ 0.0001; Pregnenolone *p* = 0.0199; 18-hydroxy-11-deoxycorticosterone *p* = 0.0039; Corticosterone *p* = 0.0003; 21-deoxycortisol *p* = 0.0153; Delta-4-androstenedione *p* = 0.0442.

RXFP2 + AngII vs Ctrl+AngII: ****p ≤ 0.0001; Pregnenolone *p* = 0.0003;18-hydroxy-11-deoxycorticosterone *p* = 0.0004; 21-deoxycortisol *p* = 0.0227.

CASZ1 vs Ctrl: ****p ≤ 0.0001; Pregnenolone *p* = 0.0083; 11-deoxycorticosterone *p* = 0.0002; 18-hydroxy-11-deoxycorticosterone *p* = 0.0162; 18-hydroxycorticosterone *p* = 0.0013; Aldosterone *p* = 0.0013.

CASZ1 + AngII vs Ctrl+AngII: ****p ≤ 0.0001; Pregnenolone *p* = 0.0057; 11-deoxycorticosterone *p* = 0.0056; 18-hydroxycorticosterone *p* = 0.0013; Aldosterone *p* = 0.0013; Delta-4-androstenedione *p* = 0.0063.

## Discussion

Our study explores the genetic susceptibility to develop PA, the most frequent form of secondary and curable arterial hypertension. Using GWAS we identify two main risk loci on chromosome 1 and 13, as well as an additional locus on chromosome 11, which are associated to PA in a French discovery cohort, replicated in a large German cohort and in a meta-analysis combining the German cohort with two smaller replication cohorts, and confirmed in the global meta-analysis including 1162 cases and 3296 controls. The locus on chromosome 13 appears to be specific to men and stronger in BAH than in APA. Two candidate genes, *CASZ1* on chromosome 1 and *RXFP2* on chromosome 13, are expressed in the adrenal gland in different cell clusters and their overexpression in adrenocortical cells significantly affects mineralocorticoid biosynthesis.

Our results suggest that CASZ1 and RXFP2 influence adrenal gland function and that risk alleles at chromosome 1 and 13 may increase susceptibility to develop PA by modifying the basal and stimulated mineralocorticoid output in the adrenal gland. Although our study is limited by the unavailability of normal adrenal tissue to test for eQTLs and to replicate those identified in GTEx, and the link between risk alleles on chromosome 1, CASZ1 and APA development remains to be established, our results suggest a biological function of CASZ1 and RXFP2 on aldosterone biosynthesis and/or adrenocortical lineage. Data on RXFP2 suggest a mechanism whereby a genetically determined reduction in basal or Ang II-stimulated aldosterone production by the zona glomerulosa leads to PA through lifelong increased stimulation of the adrenal cortex to ensure appropriate aldosterone levels. Indeed, activation of the renin-angiotensin system is a major stimulus for the expansion of the zona glomerulosa^58,59^. Such a mechanism is supported by previous results in healthy volunteers carrying hypomorphic alleles of the mineralocorticoid receptor (MR, encoded by *NR3C2*)^60^ and by the description of APA in patients with a chronically activated renin angiotensin system due to renal artery stenosis^61,62^. Under ad libitum sodium and potassium diet, healthy volunteers with different *NR3C2* rs2070951 alleles show plasma renin and aldosterone levels within the normal range, which do not differ across genotypes. When the renin-angiotensin system is challenged by moving from a high sodium-low potassium to a low sodium-high potassium diet, individuals with hypomorphic MR alleles show higher aldosterone and renin levels compared with other genotypes. This effect is attributed to less efficient sodium reabsorption because of lower levels of MR in the distal tubule of the kidney^60^. In extreme cases, adult patients with pseudohypoaldosteronism type 1 carrying heterozygous loss-of-function mutations in the MR, who are able to maintain normal sodium and potassium balance and blood pressure through increase of renin and aldosterone levels, with time develop partially autonomous aldosterone production with increased aldosterone to renin ratio compared to a control population^63^. Such a mechanism would favor the development of PA through a continuous stimulatory drive to the adrenal gland. This mechanism is also reminiscent of tertiary hyperparathyroidism, in which long-lasting secondary hyperparathyroidism in patients with chronic kidney disease may become autonomous and lead to hyperplasia or adenoma formation^64^.

A possible mechanism for this scenario could be that increased expression of RXFP2 in stem/progenitor cells or during development in carriers of risk alleles may lead to modifications of adrenal lineage and in particular of lineage conversion between the zona glomerulosa and zona fasciculata^65^. Modification of the adrenal cell phenotype could explain the diminished mineralocorticoid biosynthesis of H295R-S2 cells expressing RXFP2 and their reduced sensitivity to classic stimulators of zona glomerulosa cells such as AngII. In vivo, this may lead to a drive for replenishment of zona glomerulosa cells from adrenal cortex progenitors, which ultimately leads to adrenal cortex hyperplasia. This hypothesis is supported by the fact that RXFP2 overexpression in H295R-S2 cells increases 21-deoxycortisol levels and that patients carriers of risk alleles at RXFP2 have a trend towards lower aldosterone and higher cortisol/aldosterone ratio.

Our data also indicate that there might be sex-specific differences in susceptibility to PA. This result is not surprising when considering the well-known sex differences in adrenal cortex physiology and development of APA. Somatic *KCNJ5* mutations are more frequent in women than in men^66,67^. In mice, inactivation of *KCNK3*, coding for the potassium channel Task1, leads to a phenotype of glucocorticoid remediable aldosteronism in female only^68^. This is related to different gene expression in males and females, in particular androgen induced expression of the potassium channel Task3. Interestingly, circulating levels of INSL3, the ligand for RXFP2 are much higher in males than in females, where they follow the menstrual cycle and become undetectable after menopause^69^. The sexual dimorphism of the ligand may therefore explain the sex-dependent associations observed for RXFP2 with PA.

In conclusion, this study identifies the first risk loci for PA and suggests new mechanisms involved in adrenal gland function and the development of APA and BAH. Furthermore, it provides pathophysiological explanations for the previously observed association of *CASZ1* and *RXFP2* with blood pressure and resistant hypertension. Remarkably, those loci are in part shared between APA and BAH, in accordance with accumulating evidence for a continuum between the two conditions. Indeed, recent data showed accumulation of APCC in adrenals from patients with BAH that carry somatic mutations similar to APA^70^. Also, there are ~6% of patients undergoing adrenalectomy for lateralized aldosterone production who do not show complete biochemical cure, identifying patients with BAH with asymmetrical aldosterone production rather than unilateral APA^71^. In these patients, both solitary APA as well as hyperplasia were identified^72^ and APA carry somatic mutations similar to bona fide lateralized PA^27^. The discovery that *CASZ1* and *RXFP2* are involved in the development of PA provides new pathophysiological insight and opens perspectives for the diagnosis and treatment of arterial hypertension.

## Methods

### Patients

This research complies with all relevant ethical regulations.

#### Paris

Patients with PA were recruited within the COMETE (COrtico- et MEdullo-surrénale, les Tumeurs Endocrines) network (COMETE-HEGP protocol, CPP Ile de France II, 2012-A00508-35) or in the context of genetic screening for familial hyperaldosteronism at the Genetics department of the HEGP. Methods for screening and subtype identification of PA were performed according to institutional and the Endocrine Society guidelines^8,73,74^. In patients diagnosed with primary aldosteronism, a thin slice CT scan or MRI of the adrenal and/or an adrenal venous sampling (AVS) were performed to differentiate between unilateral and bilateral aldosterone hypersecretion. All patients gave written informed consent for genetic and clinical investigation. Procedures were in accordance with institutional guidelines. For a subset of 122 patients, somatic mutation analysis performed on fresh frozen APA tissue by whole exome or Sanger sequencing was available (50 *KCNJ5*, 23 *CACNA1D*, 7 *ATP1A1*, 4 *ATP2B3*, 5 *CTNNB1*, 1 *APC*, 32 negative^66^. For an additional subset of 42 patients with APA included in the GWAS, detailed genetic analysis by CYP11B2-immunohistochemistry guided next generation sequencing, histological information and steroid profiles were available (18 *KCNJ5*, 11 *CACNA1D*, 6 *ATP1A1*, 4 *ATP2B3*, 3 negative)^23^.

#### German cohort

Patients with PA were recruited at the Munich center of the Else Kröner-Fresenius HyperaldosteroinismusRegister—German Conn Registry (Protocol 379-10, Ethikkommission der LMU München). The diagnosis of PA was made according to the Endocrine Society Practice Guidelines^8^. The screening test consisted of a baseline plasma aldosterone-to-renin ratio (ARR; cut-off 12.0 ng/U, sitting position). If elevated, diagnosis of PA was confirmed by an abnormal confirmatory test (e.g., salt loading test, captopril challenge test or both). Antihypertensive medication was stopped before testing. If not feasible, it was replaced by the alpha 1-adrenergic receptor blocker doxazosin or calcium-channel blocker verapamil. The subtype diagnosis between unilateral and bilateral adrenal hyperplasia was based on simultanous bilateral adrenal vein sampling without ACTH stimulation as published earlier^75^. Fifty individuals were not assigned to a subtype. 127 patients had genetic analysis performed either by whole exome or Sanger sequencing. Mutation status was the following: 56 *KCNJ5*, 14 *CACNA1D*, 9 *ATP1A1*, 14 *ATP2B3*, 2 *CTNNB1*, 32 negative.

#### Italian cohort

Patients with PA (APA and BAH) were referred to the European Society of Hypertension Specialized Center of Excellence of the University of Padua, Italy. They underwent a biochemical screening for secondary causes of hypertension and provided informed written consent (Prot.1925P/2009, Comitato Etico per la Sperimentazione, Azienda Ospedaliera di Padova, Regione Veneto). They were submitted to subtype identification by adrenal vein sampling without stimulation and the diagnosis was performed following the PAPY Study^76^ and the Endocrine Society guidelines^8^. 79 patients had genetic analysis performed by targeted Sanger sequencing and 20 *KCNJ5* and 3 *CACNA1D* mutations were identified.

### Controls

The Paris Prospective Study 3 (PPS3) is an observational prospective study evaluating the role of a set of novel biomarkers on cardiovascular disease in a healthy population^77^. Briefly, the PPS3 cohort consists of 10,157 volunteers aged 50 to 75 years recruited from a large preventative medical center, the Centre d’Investigations Préventives et Cliniques in Paris (France) between June 2008 and May 2012. All participants have provided written informed consent and the study protocol was approved by the Ethics Committee of the Cochin Hospital (Paris). The study is registered in the international trial registry (URL: http://www.clinicaltrials.gov. Unique identifier: NCT00741728). Genotyping was performed on 4056 index subjects using the Illumina HumanExome-12v1.1 BeadChip as described in ref. 78.

Participants of the SUVIMAX study were healthy volunteers free of hypertension, cardiovascular disease, or cancer at baseline, recruited in metropolitan France and of European descent^79^. Genetic information was available in a subsample of 1518 participants^80^.

The Cooperative Health Research in the Region of Augsburg (KORA) cohort comprises several population-based cohort studies in the region of Augsburg, Southern Germany^81^. KORA S3 is an independent population-based sample aged 25 to 74 years that was studied between 1994 and 1995. As controls we selected 1847 subjects, who participated in a follow-up examination of S3 (KORA F3, 2004–2005) and were then persons between the ages of 35 and 84 years.

A random sample of 300 Italian normotensive controls of the HYPERGENES cohort were included^82^. A participant could be included in HYPERGENES as normotensive if he/she could self report to be of Caucasian Origin, was unrelated with other participants, had DBP < 85 mmHg and SBP < 135 at least until 55 years of age and had never been treated for hypertension. All were otherwise healthy, non obese (body mass index <30), non dyslipidemic (serum cholesterol <250; serum triglycerides <200 mg/dl—values obtained at screening prior inclusion in the study) and had no abnormal findings on physical examination. A large proportion of the sample has been followed for many years after DNA collection, allowing for the exclusion of controls that developed hypertension at a later age. Genotyping was performed on an Illumina 1 M Duo Chip; in the present study, only 1520 SNPs including 16 SNPs to replicate and Ancestry Informative Markers were available.

### Illumina genotyping of the cases in the discovery and replication cohorts

Before genotyping, a quality control was systematically performed on the samples. All samples have been quantified by fluorescence, in duplicate, using the Quant-It kits (Invitrogen). The lowest values systematically underwent a second measurement before any sample was excluded. The quality of material sent was estimated on about 10% of the total samples received (selected randomly throughout the collection) by performing: i) a quality check by migration on a 1% agarose gel to ensure the samples were not degraded, ii) a standard PCR amplification reaction on the samples to ensure that the genomic DNA was free of PCR inhibitors, iii) a PCR test to verify the sex of the individual^83^. All samples with concentrations below 20 ng/µL or having major quality problem (degradation and/or amplification problems, as well as all samples for which sex verification was discordant from the information provided) were systematically excluded from the study.

After quality control, DNAs have been aliquoted in 96-well plates (JANUS liquid handling robot, PerkinElmer) for genotyping; sample tracking was ensured by a systematic barcode scanning for each sample. Two DNA positive controls were systematically inserted in a random fashion into the plates. Genotyping was performed on Illumina OmniExpressExome8v1-2 (discovery cohort) or Illumina Global Screening Array chips (replication cohorts), on a high throughput Illumina automated platform, in accordance with the standard automated protocol of Illumina® Infinium HD Assay (Illumina ®, San Diego, USA). Several quality controls were systematically included during the process and reading of the chips was performed on iScan+ scanners (Illumina®, San Diego, USA).

Primary analysis of the results was done using the GenomeStudio software (Illumina®, San Diego, USA). The analysis of the internal controls provided by Illumina and the randomly distributed positive controls allowed the validation of the technological process

### TaqMan genotyping of the cases in the replication cohorts

For ten SNPs that we chose for the replication step but that were not available on the Global Screening Array chip, TaqMan genotyping was performed on an Applied Biosystems 7900HT Sequence detection system, according to manufacturer’s instructions. Probes were ordered from ThermoFisher-Applied biosystems. Internal tests performed prior to genotyping to validate the probes performed well. Genotyping results reading and validation were performed in duplicate by 2 different persons. The positive and negative controls included on each plate allowed the validation of the technological process. SNPs with abnormal genotyping profiles have been excluded before analysis.

### Quality control of genotyping data

Investigators of the other cohorts independently performed genotyping using different genetic platforms (Supplementary Table 1), but we included similar and standard quality-control measures to control for the quality of the genotypes.

### GWAS analysis

For each discovery or replication dataset, we applied a logistic regression (additive) model as implemented in PLINK version 1.9 (for genotype data) and version 2.0.2.3 (for imputed dosage data) (www.cog-genomics.org/plink)^84^ to test the association with PA, including as covariates the sex as well as the first ten components obtained by the multidimensional scaling method applied to the identity-by-state matrix. For SNPs on the X chromosome, we coded male pseudo-genotypes 0 or 2, instead of 0 or 1, to model X-inactivation. A genome-wide significant p-value threshold at *p* < 7.36 × 10^−8^ was considered for genotyped data. Association observed at a locus in the discovery cohort was replicated if its association was significant in the German cohort, irrespective of the global meta-analysis results. A schematic model of the multi-stage case control analyses is provided as Supplementary Figure 10.

### Imputation

For subjects of the discovery cohort, the 679,237 SNPs in common between cases and controls were phased using SHAPEIT^85^. The minimac4^86^ program was then used for the imputation of 8,332,343 SNPs and indels (estimated minor allele frequency >0.1 and estimated *r*^2^ > 0.8). The reference panel used was the 1000 Genomes Phase III (v5) panel in NCBI Build 37 (hg19). We did not impute genotypes on the replication cohorts, as the number of common SNPs between cases and controls was too low to obtain consistent imputation. A genome-wide significant p-value threshold at *P* < 5 × 10^−8^ was considered for imputed data.

### Conditional analysis

Conditional analysis was performed on imputed data for the six suggestive/significant loci identified in the discovery cohort (within 500 kb of the most associated SNP for each locus). Independent association signals were declared using a significance threshold of 10^−4^.

### Meta-analysis

The replication meta-analysis and the joint analysis of the discovery and replication stages were carried out as a fixed effect inverse-variance meta-analysis using METASOFT^87^. Moreover, in order to account for the heterogeneity between the different studies, the data was also analyzed with a random-effects model implemented in METASOFT using default settings.

### Heterogeneity analysis

Heterogeneity between men and women association results, as well as between APA and BAH association results, was tested with METASOFT as well as using the Cochran Q statistic and considered significant at *P* ≤ 7.2 × 10^−3^ (after Bonferroni correction for the seven independent loci associated in the discovery cohort).

### ATAC-seq and ChIP-seq analysis

ENCODE ATAC-seq and ChIP-seq files from adrenal gland were downloaded and visualized using pyGenometracks package (https://pygenometracks.readthedocs.io). We used the public database GENCODEv38 (https://ftp.ebi.ac.uk/pub/databases/gencode/Gencode_human/release_39/gencode.v39.annotation.gtf.gz) to retrieve genomic data. UCSC lift-over online tool (https://genome.ucsc.edu/cgi-bin/hgLiftOver) was used to convert the original assemblies used in the GWAS into GRCh38 using chromosome coordinates. Publicly available ATAC-seq and ChIP-seq datasets used in this study, accessible through the ENCODE portal (https://www.encodeproject.org/), are listed in Supplementary Table 13.

### In situ mRNA analysis and immunohistochemistry

In situ mRNA analysis was performed using the RNAscope assay^88^. For RNAscope experiments, fresh slides were cut from formalin fixed paraffin embedded adrenal tissues from 4 patients with APA and adrenals from 12 weeks old (two male and two female) C57/BL6-SC129 mice. Animal studies were conducted according to the guidelines formulated by the European Comission for experimental use (Directive 2010/63/EU) and were approved by the Institut National de la Santé et de la Recherche Médicale (Inserm), by the local Ethics comitee of Paris Descartes University (N° 17-020) and by the French Ministère de l’enseignement Supérieur, de la Recherche et de l’Innovation.

RNAscope for human and mouse *RXFP2* and *CASZ1* was performed according to the manufacturers’ instructions using the RNAscope 2.5 assay or the RNAscope 2.5 HD Duplex Detection kit (ACD, Biotechne). CYP11B2 immunohistochemistry was performed as described in^23^. In brief, sections were deparaffinised in xylene and rehydrated through graded ethanol. For antigen unmasking, the slides were incubated in Trilogy solution (1/20) (Sigma-Aldrich; St Louis, MO USA), and endogenous peroxidases were inhibited by incubation in 3% hydrogen peroxide (Sigma-Aldrich). Nonspecific staining was blocked with filtered Tris 0.1 M pH 7.4, 10% horse serum and 0.5% SDS for 60 min. The slides were incubated overnight at 4 °C with a mouse monoclonal antibody against aldosterone-synthase (hCYP11B2, clone 41-13C, 1/100, kindly provided by Dr. Celso Gomez-Sanchez), followed by 30 min incubation with anti mouse secondary antibody (1/400, Vector Laboratories; Burlingame, USA). Slides were washed and incubated with an avidin-biotin-peroxydase complex (Vectastain ABC Elite; Vector Laboratories) for 30 min, developed using diaminobenzidin (Vector Laboratories; Burlingame, USA) and counterstained with hematoxilin (Sigma-Aldrich; St Louis, MO USA).

For two patients, whose adrenals were analyzed by RNAscope experiments, snRNAseq data on the adjacent cortex were also available; characteristics of their adrenals are presented in Supplementary Figure 11.

### Single-nucleus RNA-sequencing (snRNAseq)

For snRNA sequencing experiments, a total of five pieces of adrenal cortex tissue from two adrenals with APA were collected within the COMETE-HEGP protocol. Patient 1 was a 50 ys old female with an APA carrying a *KCNJ5* p.Gly151Arg mutation; patient 2 was a 55 ys old male with an APA carrying an *ATP2B3* p.Leu425_Val427del mutation. Characteristics of patients and their adrenals are presented in Supplementary Figure 11. Tissues were immediately snap frozen in liquid nitrogen and stored at −80 °C for subsequent use. FACS sorting of nuclei and snRNAseq was performed as previously described^89^. Briefly, after lysis and homogenization, nuclei were pelleted by centrifugation for 5 min at 500 × *g* at +4 °C, washed to remove ambient RNAs, and after centrifugation stained with DAPI (10 µg/ml) during 15 min in the dark at +4 °C. After washing, nuclei were FACS sorted to exclude debris with a BD FACSAria III and the BD FACSDIVA software. After adjusting the concentration of nuclei to 1000 nuclei/μl with wash buffer, around 4000 nuclei per condition were loaded into the 10x Chromium Chip using the Single-Cell 3′ Reagent Kit v3 according to the manufacturer’s protocol. After GEM-Reverse Transcription and cDNA amplification, libraries were constructed by performing the following steps: fragmentation, endrepair, A-tailing, SPRIselect cleanup, adapter ligation, SPRIselect cleanup, sample index PCR, and SPRIselect size selection. Libraries were sequenced by pair with a HighOutput flowcel on an Illumina Nextseq 500. A minimum of 46,000 reads per nucleus (50,000 targeted) were sequenced and analyzed with Cell Ranger Single-Cell Software Suite 3.0.2 by 10x Genomics. Data analysis was performed using the GRCh38 reference genome built provided by 10x Genomics on their Support website^89^. The subsequent visualizations, clustering and differential expression tests were performed in R (v 3.4.3) using Seurat (v3.0.2).

### Functional studies in H295R-S2 cells

The human adrenocortical carcinoma cell line H295R strain 2 (H295R-S2), kindly provided by W. E. Rainey^90^ was cultured in DMEM/Eagle’s F12 medium (GIBCO, Life technologies, Carlsbad, CA) supplemented with 2% Ultroser G (PALL life sciences, France), 1% insulin/transferrin/selenium Premix (GIBCO, Life technologies, Carlsbad, CA), 10 mM HEPES (GIBCO, Life technologies, Carlsbad, CA), 1% penicillin, and streptomycin (GIBCO, Life technologies, Carlsbad, CA) and maintained in a 37 °C humidified atmosphere (5% CO_2_).

For overexpression experiments, H295R-S2 cells were seeded into tissue culture dishes 100 at a density of 5.000.000 cells per dish, and maintained in the conditions described. After 24 h, cells were resuspended in 100 μl Nucleofector R solution (AMAXA kit, Lonza) and transfected with 3 μg of pcDNA3 containing the RXFP2 or CASZ1 cDNA or an empty pcDNA3 vector, using the electroporation program P-20. To select only stably transfected cells, 48 h post transfection cells were changed to medium containing 500 µg/mL G418-Genetycin (Gibco) and used after all untransfected cells had died. G418 selection was kept during all functional studies. For aldosterone measurements and RNA extraction, cells were serum deprived in DMEM/F12 containing 0.1% Ultroser G for 24 h and then incubated for another 24 h with fresh medium containing 0.1% Ultroser G with no secretagogue (basal) or 10 nM of AngII (A-II 10 nM). At the end of this incubation time, supernatant and cells from each well were harvested for aldosterone measurement and protein extraction. Two experiments were independently conducted in quadruplicate.

### RNA extraction and RT-qPCR

Total RNA was extracted in Trizol reagent (Ambion Life technologies, Carlsbad CA) according to the manufacturer’s recommendations. After deoxyribonuclease I treatment (Life Technologies, Carlsbad, CA), 500 ng of total RNA were retrotranscribed (iScript reverse transcriptase, Biorad, Hercules, CA). Primer pairs for *RXFP2* and *CASZ1* used for qPCR are qHsaCID0036539 and qHsaCID0014926 (Biorad, Hercules, CA). Quantitative PCR was performed using SYBRgreen (Sso advanced universal SyBr Green supermix, Biorad, Hercules, CA) on a C1000 touch thermal cycler of Biorad (CFX96 Real Time System), according to the manufacturer’s instructions. Controls without template were included to verify that fluorescence was not overestimated from primer dimer formation or PCR contaminations. RT-qPCR products were analyzed in a post amplification fusion curve to ensure that a single amplicon was obtained. Normalization for RNA quantity, and reverse transcriptase efficiency was performed against three reference genes (geometric mean of the expression of Ribosomal *18S* RNA, *HPRT*, and *GAPDH*), in accordance with the MIQE guidelines^91^; primers are described in Supplementary Table 14^19^. Quantification was performed using the standard curve method. Standard curves were generated using serial dilutions from a cDNA pool of all samples of each experiment, yielding a correlation coefficient of at least 0.98 in all experiments.

### Western blot

H295R-S2 cells were lysed using RIPA buffer (Bio Basic Canada Inc.) with protease and phosphatase inhibitors mini tablets, EDTA free (Thermo Scientific). Proteins were solubilized for 30 min at 4 °C, under end-over-end rotation, and then centrifuged at 13,000 rpm for 15 min at 4 °C. Protein concentration was determined using Bradford protein assay (Biorad). 15 µg of proteins were submitted to 10% SDS-PAGE and transferred onto nitrocellulose membrane. Membranes were blotted with the following antibodies: mouse anti-aldosterone synthase antibody (1:500, clone CYP11B2-41-13, kindly provided by Dr C Gomez Sanchez^92^ and mouse anti-tubulin (T9026, 1:2000, Sigma Aldrich). Signals were developed by Clarity Max™ Western ECL substrate (Biorad, Hercules, CA) and detected by Fujifilm Las-4000 mini Luminescent image analyzer (Fujifilm, Tokyo-Japan) and quantified by Multi gauge software (Fujifilm, Tokyo-Japan). Expression of total proteins was normalized to the expression of the housekeeping protein tubulin. Uncropped and unprocessed scans of blots are presented in the Source Data file.

### Liquid chromatography coupled to tandem mass spectrometry steroid profiling

Fourteen steroids are measured simultaneously by liquid chromatography coupled to tandem mass spectrometry: pregnenolone, progesterone, 11-deoxycorticosterone, corticosterone, 18-hydroxy-11-deoxycorticosterone, 18-hydroxycorticosterone, aldosterone, 17-hydroxyprogesterone, 21-deoxycortisol, 11-deoxycortisol, cortisol, 18-hydroxycortisol, 18-oxocortisol, delta-4-androstenedione in a 13-min run. The complete steroid profiling procedure is described in ref. 23.

### Statistical analyses

Quantitative variables are reported as means ± SEM when Gaussian distribution or medians and interquartile range when no Gaussian distribution. Pairwise comparisons were done with unpaired *t* test or Mann-Whitney test respectively; global comparison was evaluated using ANOVA or Kruskall Wallis test, followed by Sidak’s or Dunn’s multiple comparison test. A *p* value < 0.05 was considered significant for comparisons between two groups. Analyses were performed using Graphpad Prism 9 (GraphPad software Inc, San Diego, CA) or MedCalc19 (MedCalc software Ltd).

### Reporting summary

Further information on research design is available in the Nature Research Reporting Summary linked to this article.

## Supplementary information

Supplementary Information

Description of Additional Supplementary Files

Supplementary Data 1

Supplementary Data 2

Supplementary Data 3

Supplementary Data 4

Reporting Summary

## Data Availability

Summary statistics that support the findings of this GWAS has been deposited in the GWAS catalog database under accession codes GCST90129615, GCST90129616, GCST90129617, GCST90129618, GCST90129619, GCST90129620, GCST90129621, GCST90129622, GCST90129623, GCST90129624 [https://www.ebi.ac.uk/gwas/]. The individual level genotype data will not be publicly available since they contain information that could compromise research participant privacy and consent. snRNAseq data have been deposited in the NCBI Gene Expression Omnibus (GEO) database [https://www.ncbi.nlm.nih.gov/geo/] under accession code GSE210381. ATAC-seq and ChIP-seq datasets used in this study were retrieved from public databases and a full list of files with accession is available in Supplementary Table 13. Accession details to publicly available gene expression datasets used for Supplementary Fig. 8 are mentioned in the figure legend. All data supporting the findings of this study are available within the manuscript and supplementary information/Source Data file or from the corresponding author upon reasonable request. Source data are provided with this paper.

## Source data

Source Data

## Code availability

Publicly available softwares and packages were used throughout this study as described in the Methods section. No custom algorithms were generated for the study.
