# Supplementary material for: Identification of risk loci for primary aldosteronism in genome-wide association studies
Source: Nat Commun. 2022 Sep 3;13:5198. doi: 10.1038/s41467-022-32896-8 (PMC9440917; doi:10.1038/s41467-022-32896-8)
Supplement: Supplementary file 1 — Supplementary Information [file 41467_2022_32896_MOESM1_ESM.pdf]

Identification of risk loci for primary aldosteronism in genome-wide association studies

Edith Le Floch[1*], Teresa Cosentino[2*], Casper K. Larsen[2], Felix Beuschlein[3,4], Martin Reincke[3], Laurence Amar[2,5], Gian-Paolo Rossi[6], Kelly De Sousa[2], Stéphanie Baron[7,8], Sophie Chantalat[1], Benjamin Saintpierre[9], Livia Lenzini[6], Arthur Frouin[1], Isabelle Giscos-Douriez[2], Matthis Ferey[2], Alaa B. Abdellatif[2], Tchao Meatchi[2,10], Jean-Philippe Empana[2], Xavier Jouven[2,11], Christian Gieger[12,13,14], Melanie Waldenberger[12,13,15], Annette Peters[13,14,15], Daniele Cusi[16,17], Erika Salvi[18], Pierre Meneton[19], Mathilde Touvier[20], Mélanie Deschasaux[20], Nathalie Druesne-Pecollo[20], Sheerazed Boulkroun[2], Fabio L Fernandes-Rosa[2], Jean-François Deleuze[1], Xavier Jeunemaitre[2,21], Maria-Christina Zennaro[2,21#]

Affiliations:

[1] Centre National de Recherche en Génomique Humaine, Institut de biologie François Jacob, CEA, Université Paris-Saclay, Evry, France

[2]Université Paris Cité, Inserm, PARCC, F-75015 Paris, France

[3]Medizinische Klinik und Poliklinik IV, Ludwig-Maximilians-University, 80336 Munich, Germany

[4]Klinik für Endokrinologie, Diabetologie und Klinische Ernährung, Universitätsspital Zürich (USZ) und Universität Zürich (UZH), Zürich, Switzerland

[5]Assistance Publique-Hôpitaux de Paris, Hôpital Européen Georges Pompidou, Unité Hypertension artérielle, Paris, France

[6]DMCS ‘G. Patrassi’ University of Padova Medical School, University Hospital, 35126 Padova, Italy

[7]Université Paris Cité, F-75006 Paris, France

[8]Assistance Publique-Hôpitaux de Paris, Hôpital Européen Georges Pompidou, Service de Physiologie, Paris, France

[9] Université Paris Cité, Institut Cochin, Genom'IC platform, INSERM, CNRS, 75014 Paris, France

[10]Assistance Publique-Hôpitaux de Paris, Hôpital Européen Georges Pompidou, Service d’Anatomie Pathologique, Paris, France

[11]Assistance Publique-Hôpitaux de Paris, Hôpital Européen Georges Pompidou, Service de Cardiologie, Paris, France

[12]Research Unit of Molecular Epidemiology, Helmholtz Zentrum München, German Research Center for Environmental Health, Neuherberg, Germany.

[13]Institute of Epidemiology, Helmholtz Zentrum München, German Research Center for Environmental Health, Neuherberg, Germany.

[14]German Center for Diabetes Research (DZD), Neuherberg, Germany.

[15]German Research Center for Cardiovascular Research (DZHK), Partner Site Munich Heart Alliance, Munich, Germany.

[16]Institute of Biomedical Technologies National Research Council of Italy, Milan, Italy

[17]Bio4Dreams-Business Nursery for Life Sciences, Milan, Italy

[18]Neuroalgology Unit Fondazione IRCCS Istituto Neurologico 'Carlo Besta' Milan Italy

[19]UMR_1142, INSERM, Sorbonne Université, Université Paris 13, Paris, France

[20]Sorbonne Paris Nord University, INSERM U1153, INRAe U1125, CNAM, Nutritional Epidemiology Research Team (EREN), Epidemiology and Statistics Research Center – Université Paris Cité (CRESS), 93017 Bobigny, France

[21]Assistance Publique-Hôpitaux de Paris, Hôpital Européen Georges Pompidou, Service de Génétique, Paris, France
*These authors contributed equally to this work
[#]Corresponding author

Address correspondence to:
Maria-Christina Zennaro, MD, PhD
INSERM, U970
Paris Cardiovascular Research Center – PARCC
56, rue Leblanc,
75015 Paris – France
Tel : +33 (0)1 53 98 80 42
Fax : + 33 (0)1 53 98 79 52
e-mail: maria-christina.zennaro@inserm.fr

Supplementary Figures and Tables

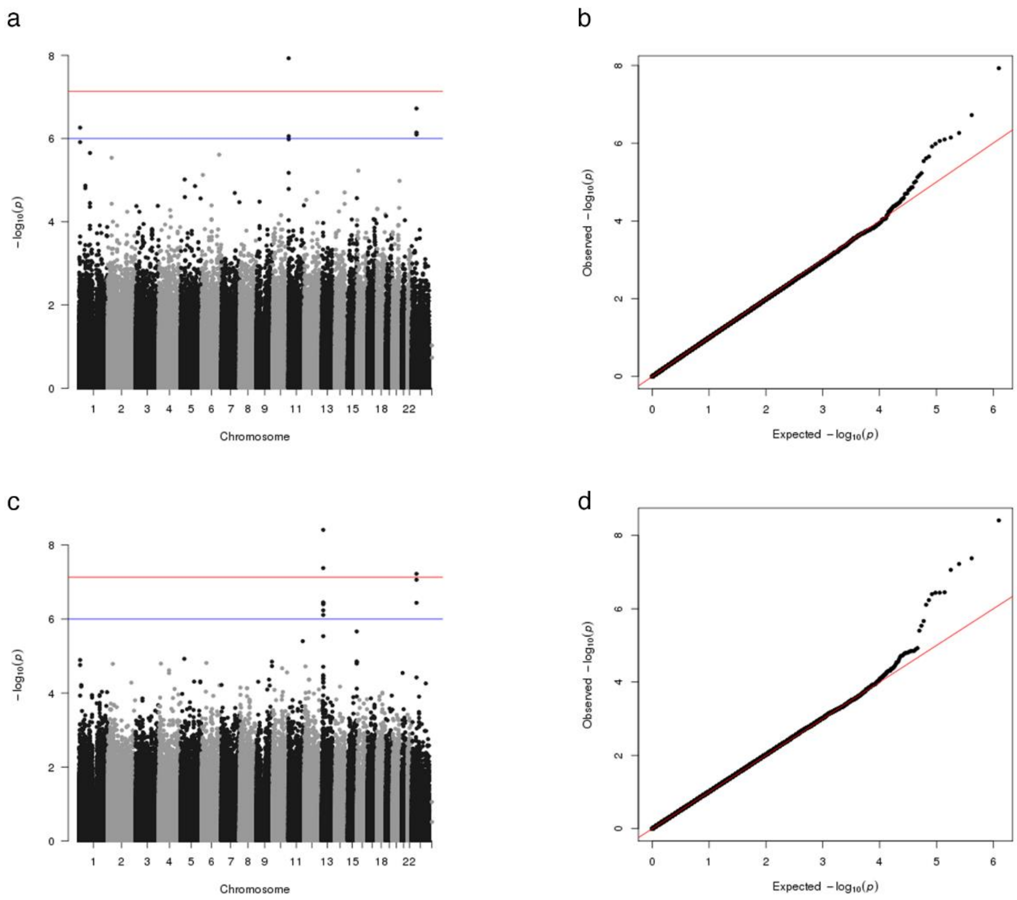

Supplementary Figure 1. Manhattan plots and quantile-quantile (QQ) plots showing results of subanalyses for APA and BAH in the discovery cohort. For APA, the locus on chromosome 11 showing genome-wide suggestive association with all PA cases showed also genome-wide significant association with APA, while the loci on chromosomes 1 and X showed suggestive association (a, b). For BAH, genome-wide significant association was found with the locus on chromosome 13 and the locus on chromosome X (c,d).

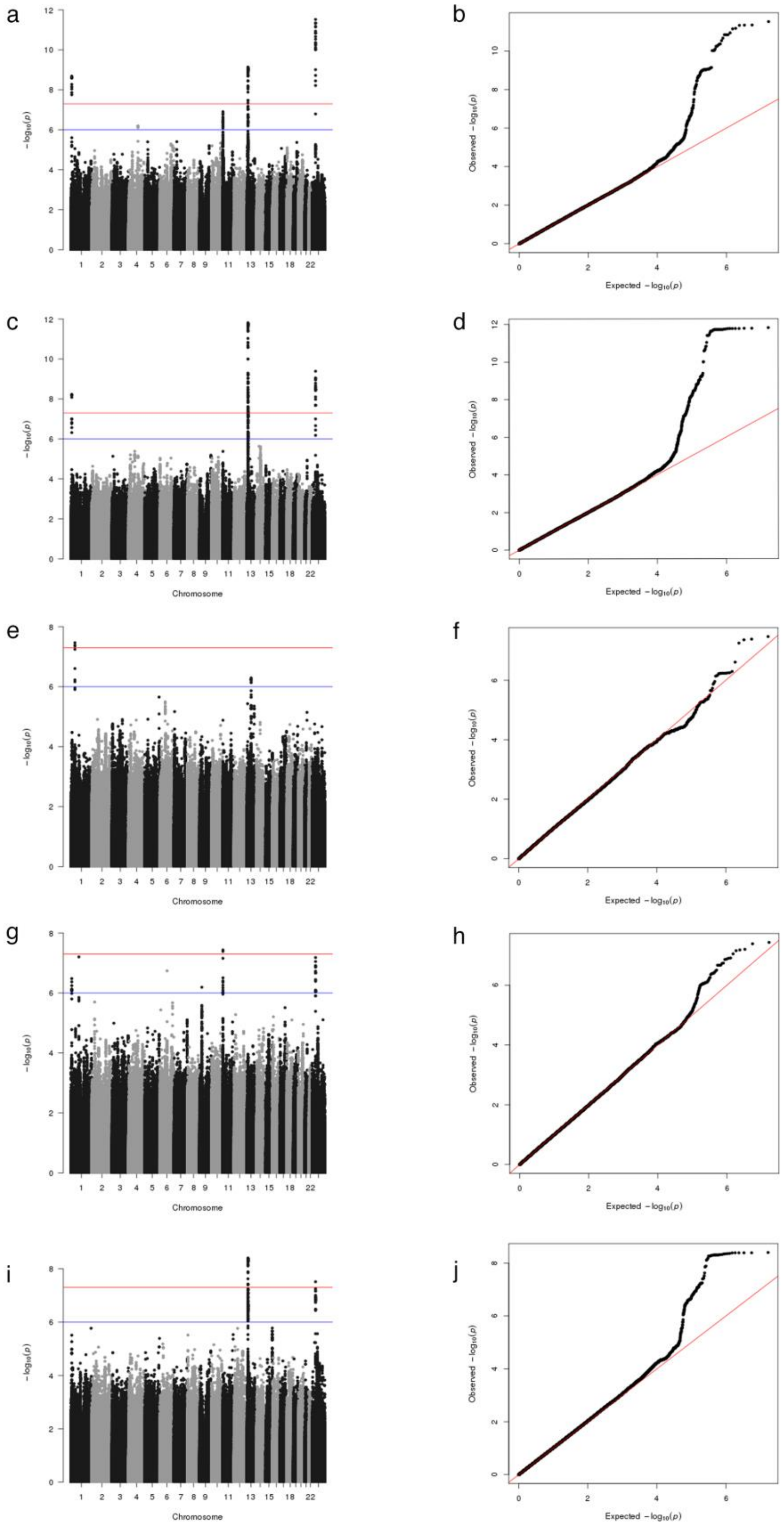

Supplementary Figure 2. Manhattan plots and quantile-quantile (QQ) plots showing results of fine mapping using imputed data in the discovery cohort. For the entire cohort, the loci on chromosomes 1, 13 and X showed genome-wide significant associations while a suggestive association was observed for the

locus on chromosome 11 (a,b). For the analysis in men, the same three loci on chromosomes 1, 13 and X showed genome-wide significant associations (c,d). For the analysis in women, the second locus on chromosome 1 showed a significant association, while the second locus on chromosome 13 showed a suggestive association (e,f). For APA, the locus on chromosome 11 showing genome-wide suggestive association with all PA cases showed also genome-wide significant association with APA, while the loci on chromosomes 1 and X showed suggestive association (g, h). For BAH, genome-wide significant association was found with the locus on chromosome 13 and the locus on chromosome X (i,j).

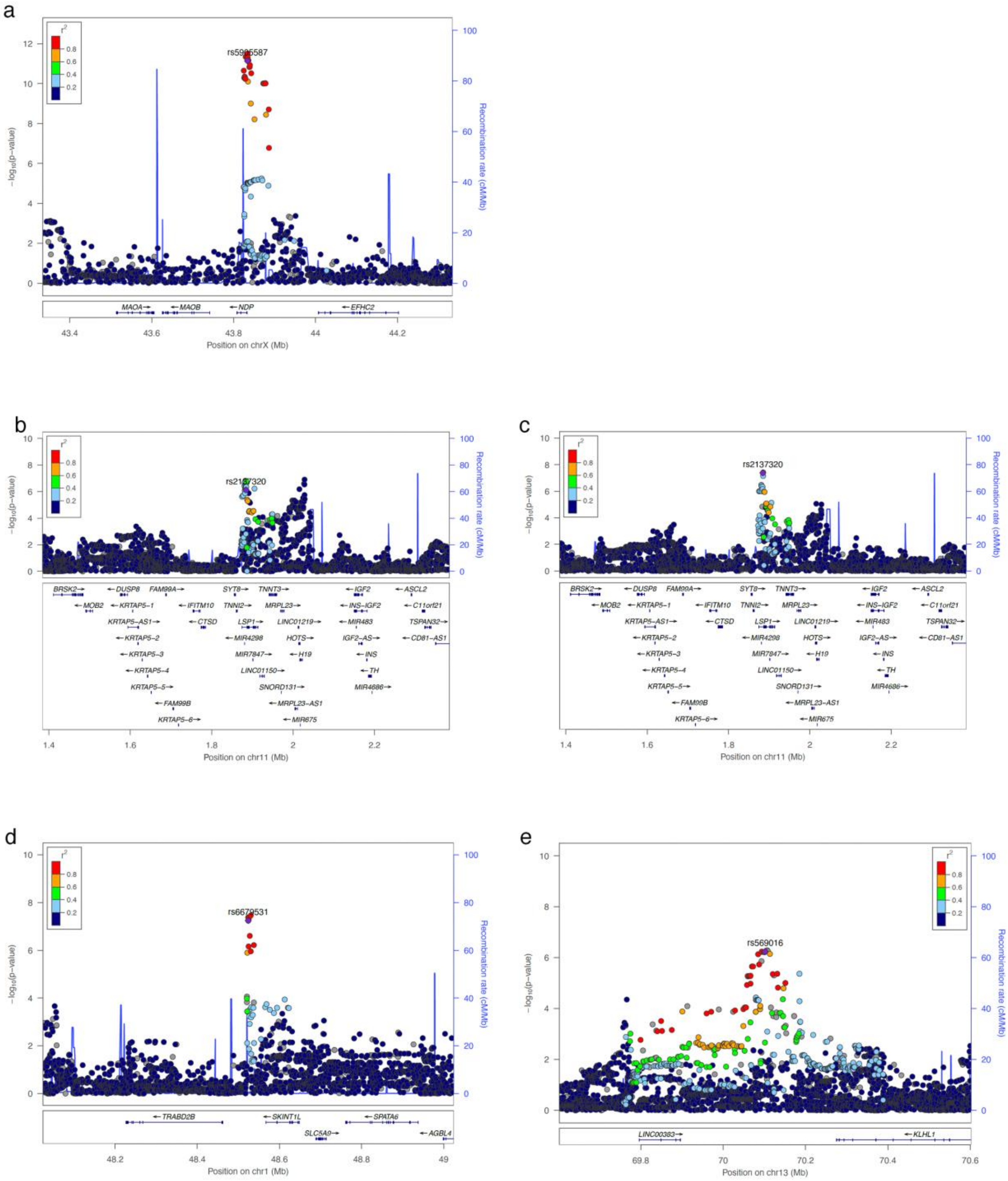

Supplementary Figure 3. Genomic context of the association signals observed in the GWAS in the discovery cohort after imputation. The regional association plots were generated using LocusZoom and display surrounding genes. The locus on chromosome X associated with the entire cohort (a) as well as loci associated with subanalyses are represented. (b-c) Locus on chromosome 11 showing suggestive association with PA (b) or significant association with APA (c). Two additional loci on chromosome 1 (d) and 13 (e) identified in women in the discovery cohort are represented. Dot color indicates linkage disequilibrium of each variant with the highlighted lead variant in common between discovery and replication cohorts (purple diamond).

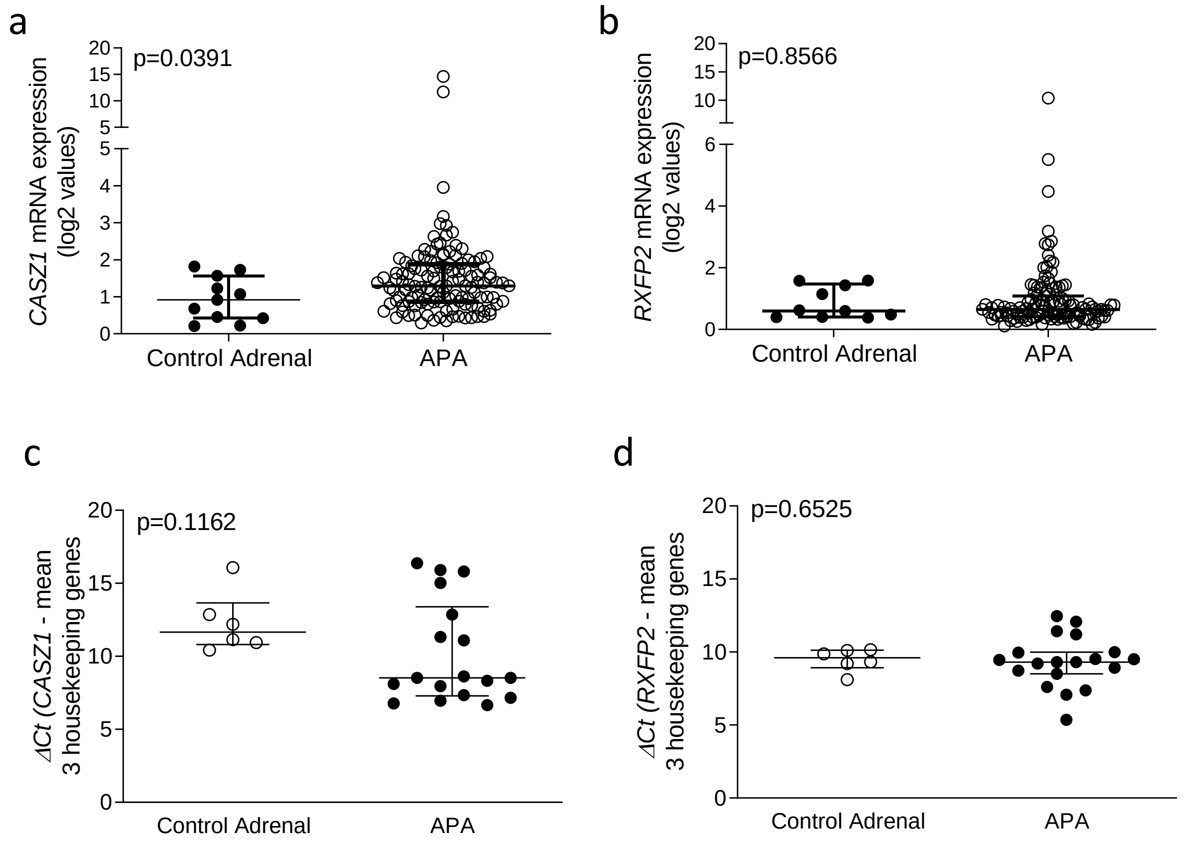

Supplementary Figure 4. Expression of candidate genes in control adrenals and APA. (a-b) mRNA expression of *CASZ1* (a) and *RXFP2* (b) in control adrenals (n=11 biological independent samples) and in APA (n=123 biological independent samples) was retrieved from a transcriptome study. Values are median centred, log2-transformed and model-adjusted expression levels and represent medians with interquartile range (two-sided Mann-Whitney test). (c-d) mRNA expression of *CASZ1* (c) and *RXFP2* (d) in control adrenals (n=6) and in APA (n=18/19) measured by RT-qPCR. Values represent ΔCt of the gene of interest – mean of three housekeeping genes (median with IQR, two-sided Mann-Whitney test).

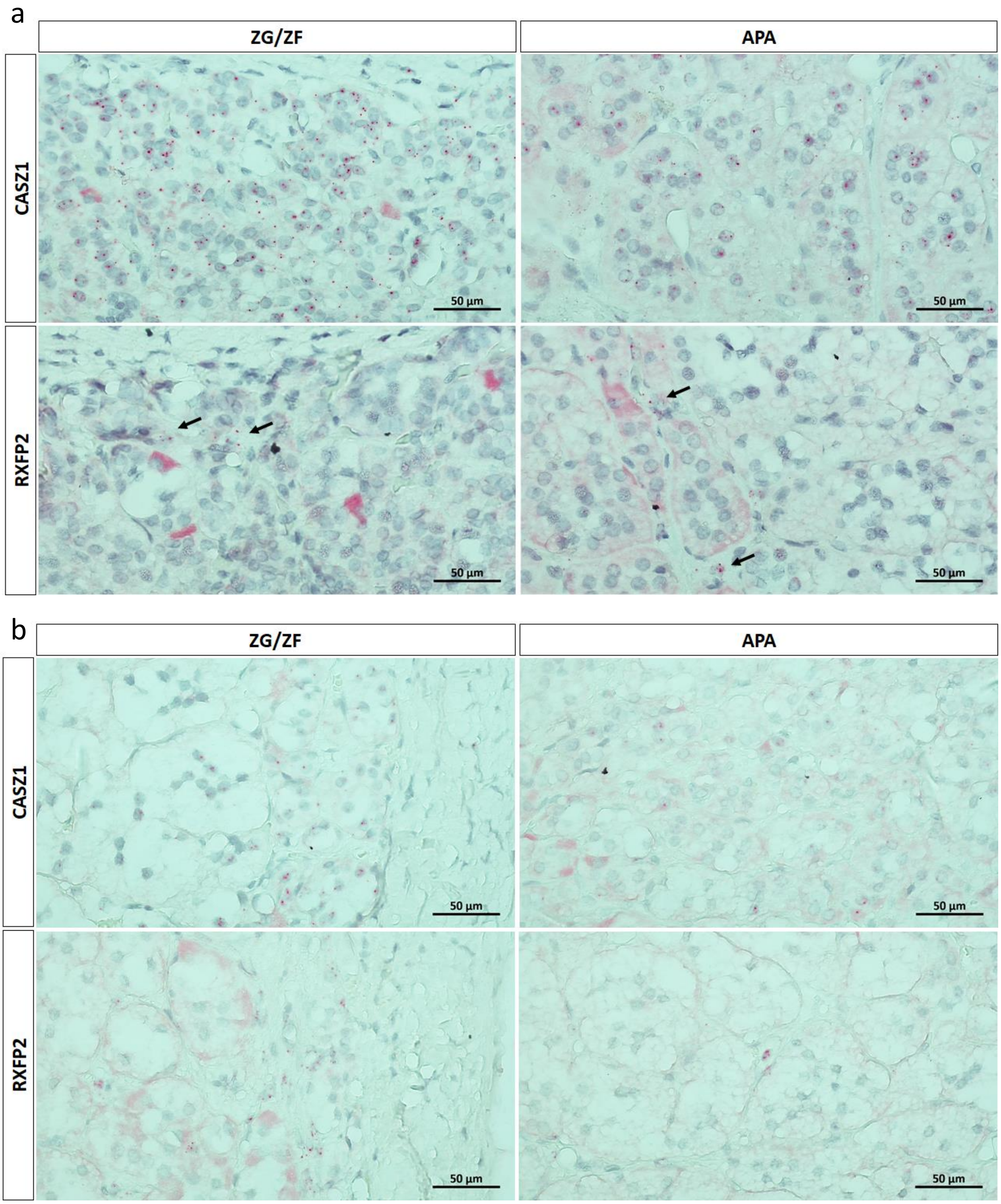

Supplementary Figure 5. Expression of CASZ1 and RXFP2 in adrenals with APA. CASZ1 and RXFP2 mRNA localization in the adrenal cortex of adrenals with APA was analysed by RNAscope. (a) adrenal with APA carrying a somatic *ATP2B3* mutation. (b) adrenal with APA negative for somatic mutations in known genes. Images are representative of results obtained in adrenals from four patients. Red dots represent positive staining. Arrows indicate positive staining. For each sample, one experiment was performed.

a

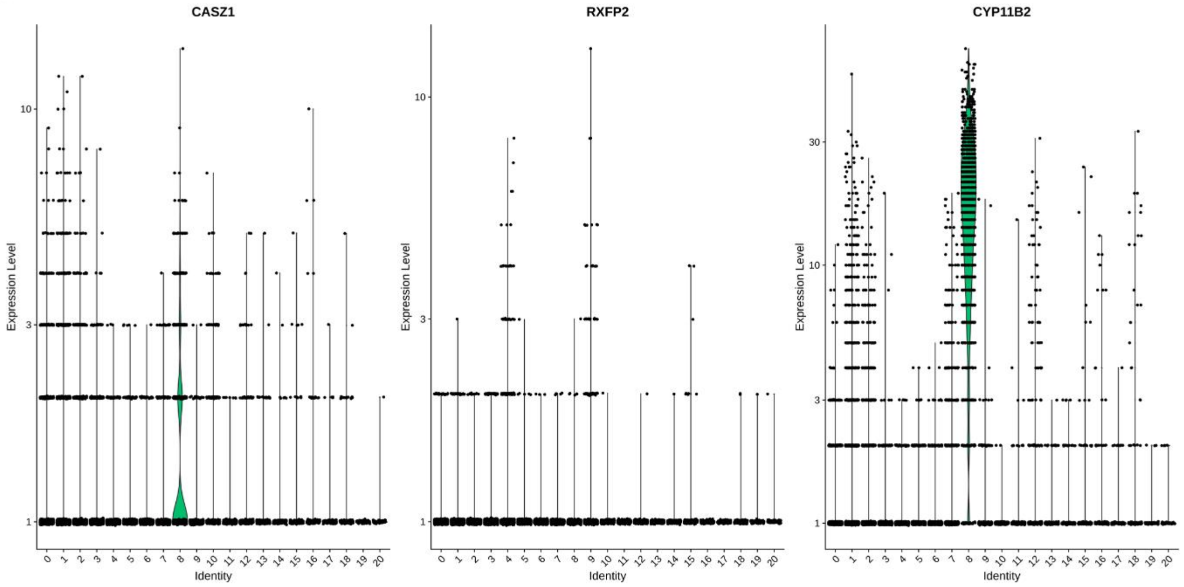

b

Cell type	Marker genes	references
ZG cells	VSNL1, DACH1, ATP10A, GABBR2, DNER, EZR, PAPSS2, ADGRV1	Zhou et al, 2016 [1]
APCC	CYP11B2, CLRN1, ALPK3, MC2R, TSPAN12, CACNB2, HTR4	Nishimoto et al, 2015 [2]
Adrenal stem/progenitor cells*	Ptch1, AFF3, LGR4, RSPO3, COL4A2, COL4A1, COL4A4, CDH2, ADGRV1, DCN, PAPSS2, DACH1, AGTR1	Pignatti et al, 2017 [3]<br>Zhou et al, 2016 [1]

Supplementary Figure 6. Expression of CASZ1 and RXFP2 in the adrenal cortex adjacent to APA. (a) Violin plots from snRNA-seq from adrenal cortex adjacent to APA showing the expression of *CASZ1*, *RXFP2* and *CYP11B2* in the different clusters. (b) List of marker genes identifying Cluster 8 as ZG/APCC cells and clusters 4 and 9 as stem/progenitor cells. *In clusters 4 and 9, a few genes defining ZG cells have also been identified.

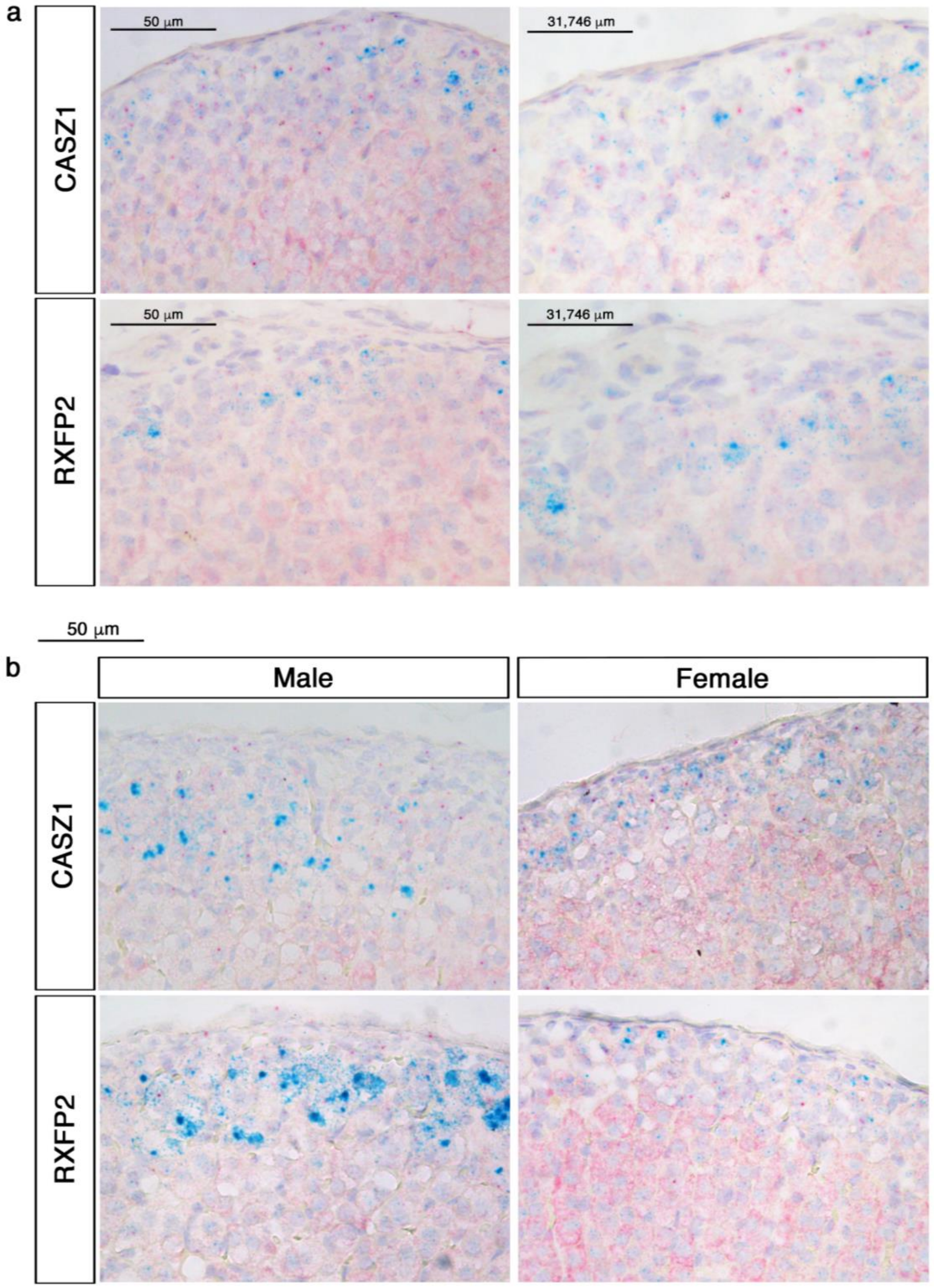

Supplementary Figure 7. Expression of Casz1 and Rxfp2 in adrenals from male and female mice. (a) *Casz1* and *Rxfp2* mRNA expression in adrenal glands from male mice analysed by duplex RNAscope. Red dots represent positive staining; blue dots represent *CYP11B2* staining. (b) *Casz1* and *Rxfp2* mRNA localization in male and female mice analysed by duplex RNAscope. Red dots represent positive staining; blue dots represent *CYP11B2* staining. Images are representative of results obtained in adrenals from two male and two female mice. For each sample, one experiment was performed.

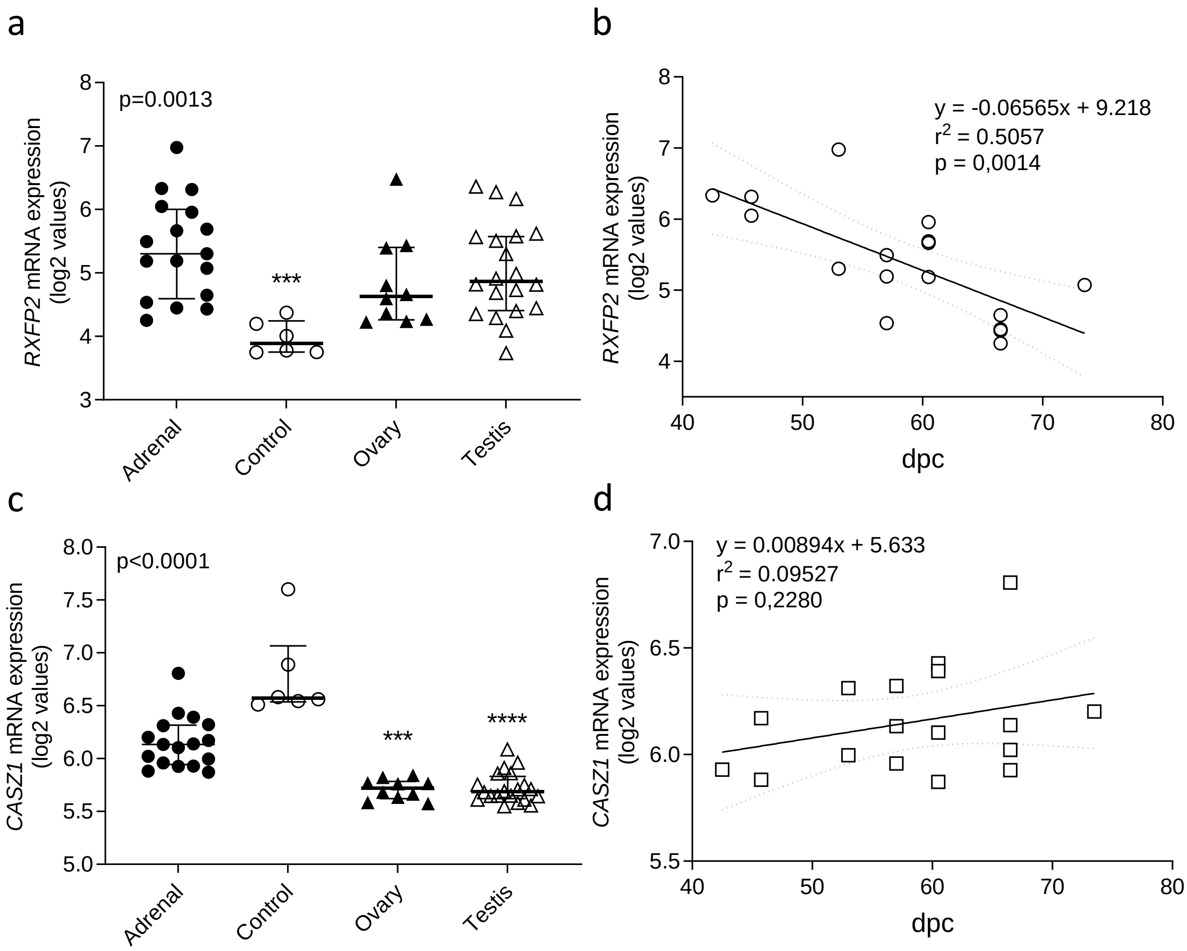

Supplementary Figure 8. Expression of RXFP2 and CASZ1 during adrenal development. Gene expression data representing normalised log2 gene expression levels of 53 samples were retrieved from [4] and reanalysed (Array data are available from the ArrayExpress database under the accession number E-MTAB-5525). *RXFP2* (a) and *CASZ1* (c) mRNA expression in adrenal gland (n=17), control tissue (n=6, from spine, brain, muscle, heart, kidney and liver), ovary (n=10) and testis (n=20). *RXFP2* (b) and *CASZ1* (d) expression between approximately 42.5 and 74 dpc. Values represent medians ± IQR. p-values are calculated using Kruskal-Wallis test, followed by Dunn's test for multiple comparisons. *CASZ1*, Adrenal vs. Ovary: ***, p=0,0007; Adrenal vs. Testis : ****, p<0.0001; *RXFP2*, Adrenal vs. Control: ***, p=0,0003. dpc, days post conception.

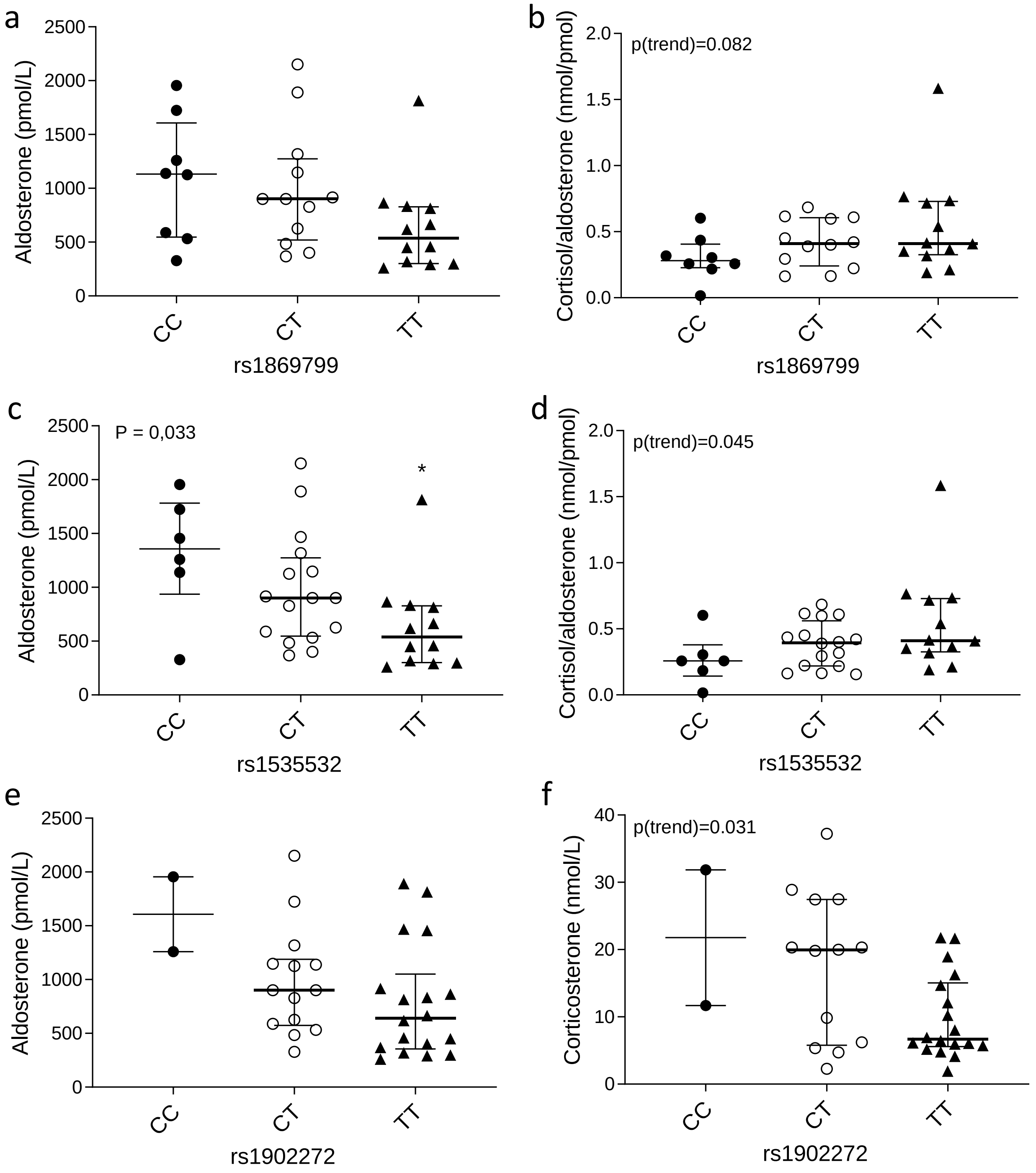

Supplementary Figure 9. Correlation of genotypes with steroid output in patients with APA. Association of genotypes for rs1869799 (a,b, n=32), rs1535532 (c,d, n=34) and rs1902272 (e,f, n=34) on chr 13 with plasma aldosterone (a, c, e), cortisol/aldosterone ratio (b,d) and corticosterone (f) levels. Values represent medians and interquartile range. p-values are calculated using ANOVA or Kruskal-Wallis test. *, p= 0,0302 versus CC. p(trend), p value by Jonckheere-Terpstra trend test.

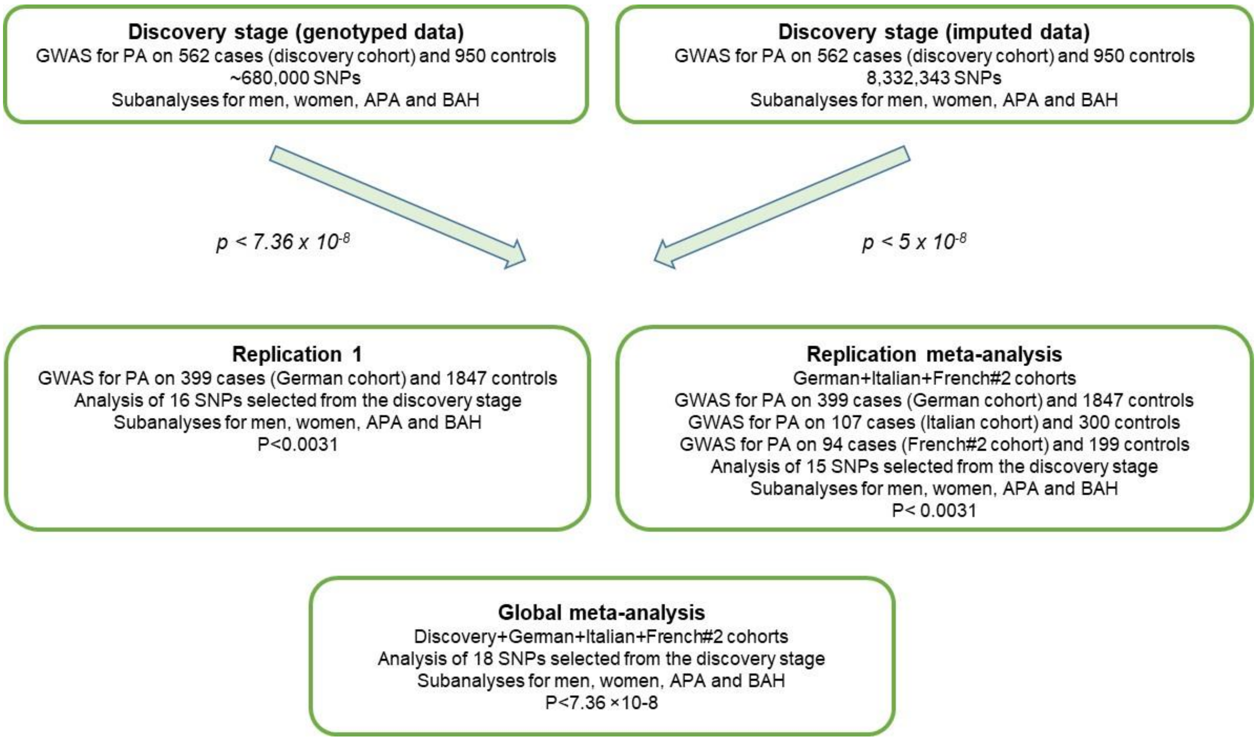

Supplementary Figure 10. Schematic model of the multi-stage case control analyses used in this study.

a

Patient	Sex	Structure	CYP11B2 staining	NGS-Mutated Gene (FFPE)	c.DNA	Protein
1	Female	APA	Yes	*KCNJ5*	c.451G>C	p.Gly151Arg
		APCC1	Yes	*Neg*		
		APCC2	Yes	*Neg*		
2	Male	APA	Yes	*ATP2B3*	c.1273_1278delCTGGTC	p.Leu425_Val427del

b

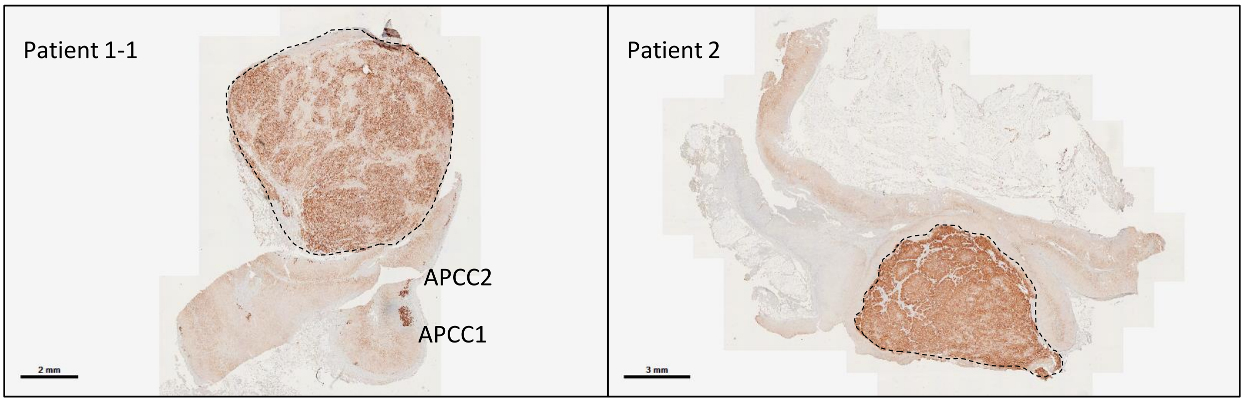

c

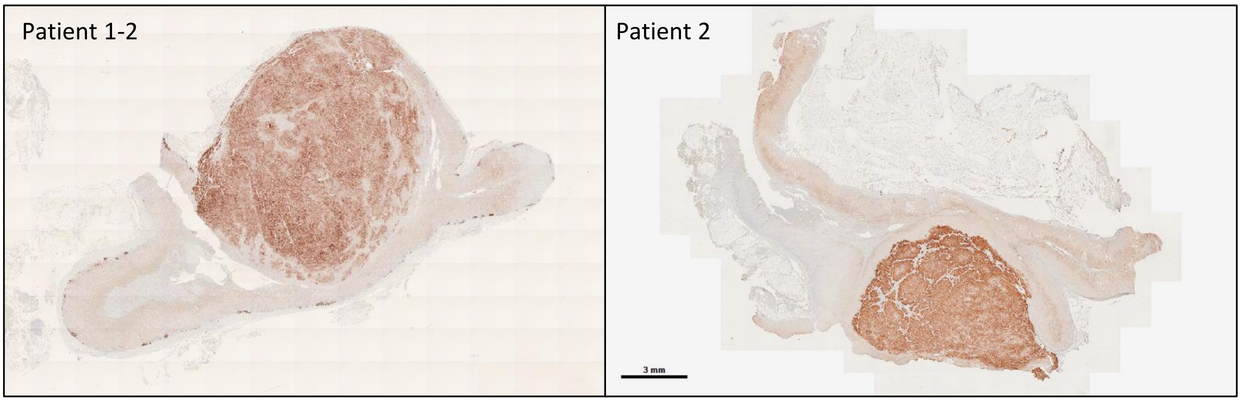

Supplementary Figure 11. Characteristics of adrenals with APA used for snRNAseq and RNAscope. (a) Patients' characteristics and somatic mutations identified by immunohistochemistry-guided NGS in APA and APCC. Neg, Negative. (b) CYP11B2 immunohistochemistry of the two adrenals. (c) CYP11B2 immunohistochemistry of consecutive slides of the same blocs used for RNAscope experiments presented in Figure 3 (patient 1, Figure 3, panel c) and Supplementary Figure 5 (patient 2, Supplementary Figure 5, panel a). For each sample, one immunohistochemistry was performed.

Supplementary Table 1. Summary of patients' characteristics and procedures of individual studies

	French discovery dataset		German replication dataset		Italian replication dataset		French replication dataset	
Study	HEGP 1st set of cases	EPP3 controls	German cases	KORA controls	Italian cases	Italian controls	HEGP 2nd set of cases	SUVIMAX controls
Number of individuals (PA subtype)	n=562 (321 APA, 240 BAH)	n=950	n=399 (214 APA, 135 BAH)	n=1847	n=107 (76 APA, 31 BAH)	n=300	n=94 (64 APA, 30 BAH)	n=199
Number of men/women	339 men 223 women	639 men 311 women	255 men 144 women	915 men 932 women	66 men 41 women	186 men 114 women	59 men 35 women	126 men 73 women
Mean age ±sd	61.6 ±13.2	65.3 ±6.4	59.4 ±12.3	46.22 ±11.92	59.3 ±11.5	60.9 ± 6.5	51.7 ±9.7	50.4 ±5.9
Call rate by individual	Individuals with a call rate <0.99 were removed							
Individual exclusions	Outliers on the first 2 components of the multi-dimensional scaling plot (PLINK) were removed. Individuals with a PI HAT (proportion of IBD) >0.125 with another individual were removed.		Outliers on the first 2 components of the Principal component analysis plot (PLINK) were removed. Individuals with a PI HAT (proportion of IBD) >0.125 with another individual were removed.					
Platform	OmniExpress Exome 8v1-2 chip	OmniExpress 24v1-0 chip	GSA + 10 SNPs by Taqman	Illumina Human Omni 2.5-4	GSA + 10 SNPs by Taqman	1520 SNPs of Illumina 1M Duo	GSA + 10 SNPs by Taqman	317k-Illumina chip
SNP call rate	SNPs with a call rate <0.99 were removed							
Hardy-Weinberg Equilibrium (HWE)	SNPs deviating from HWE in controls with a p-value <10^{-5} were removed		SNPs deviating from HWE in controls with a p-value <10^{-4} were removed					
Number of SNPs filtered in	679237 SNPs in common between arrays after QC		282196 SNPs in common between arrays after QC		1358 SNPs in common between arrays after QC		46909 SNPs in common between arrays after QC	

Supplementary Table 2. Test of heterogeneity between men and women for significant/suggestive associations with PA

rs	chr	pos	P(Q) French#1	P(Q) German	P(Q) Italian	P(Q) French#2	P(Q) Meta-analysis
**rs284277**	1	10790797	0.0889252	0.241945	0.474406	NA	0.869522
**rs880315**	1	10796866	0.386914	0.462867	0.870572	0.656435	0.97921
**rs6679531**	1	48524180	**0.000150299**[a]**	0.51162	**0.00113307**[a]**	NA	0.270764
**rs12080303**	1	48527879	**0.000938559****	NA	NA	NA	NA
**rs587961**	11	1881256	NA	0.47706	NA	NA	NA
**rs2137320**	11	1884342	0.077347	0.387716	0.665727	0.651485	0.149612
**rs4980379**	11	1888614	0.251853	0.445736	0.501778	NA	0.136947
**rs661348**	11	1905292	0.55173	NA	0.520402	NA	0.422846
**rs277129**	13	32095644	0.0304785	NA	NA	NA	NA
**rs563854**	13	32101568	0.0135036	**1.32367E-06****	0.0610787	NA	**6.24573E-08****
**rs1869799**	13	32105351	**0.000428409****	NA	NA	NA	NA
**rs1200470**	13	32112348	0.0250357	NA	NA	NA	NA
**rs1535532**	13	32114398	**0.000160566****	**3.47979E-07****	0.0278632	0.577098	**5.68165E-11****
**rs1902272**	13	32125315	**0.0044328****	NA	0.271	0.502714	**0.00225967****
**rs277143**	13	32132567	0.0158407	NA	NA	NA	NA
**rs1671966**	13	32185002	**0.00039837****	**0.000295886****	0.0344394	NA	**3.91865E-08****
**rs1327981**	13	32197584	**0.00463002****	NA	NA	NA	NA
**rs1327980**	13	32200557	**0.00329299****	**1.86376E-06****	0.0786466	NA	**1.39511E-08****
**rs9603395**	13	32202333	**6.91625E-05****	NA	NA	NA	NA
**rs2025908**	13	32226399	**0.00146247****	**4.55655E-06****	0.329696	NA	**4.89928E-08****
**rs1571303**	13	32250858	**0.000833658****	NA	NA	NA	NA
**rs569016**	13	70102087	**1.73387E-05****	0.446993	0.974861	NA	**0.00156296****
**rs1005002**	X	43827064	0.944634	0.28562	NA	NA	0.356705
**rs5905587**	X	43833996	0.784202	0.540124	NA	0.129064	0.991882
**rs5906426**	X	43877475	0.681044	0.356594	NA	0.168715	0.879068
**rs5906430**	X	43879037	0.617780	0.0893214	NA	NA	0.310538

All SNPs listed here showed a significant or a suggestive association with the phenotype in the full discovery cohort and/or in at least one stratified analysis. Heterogeneity was tested with METASOFT as well as using the Cochran Q statistic. ** significant heterogeneity after a Bonferroni correction for the 7 independent loci (P<0.0072) in bold.[a] Please note that these two p-values do not reflect a heterogeneity in the same direction (see Table 1 and Supplementary Table 5).

Supplementary Table 3. Test of heterogeneity between APA and BAH for significant/suggestive associations with PA

rs	chr	pos	P(Q) French#1	P(Q) German	P(Q) Italian	P(Q) French#2	P(Q) Meta-analysis
**rs284277**	1	10790797	0.983066	0.207259	0.538195	NA	0.501042
**rs880315**	1	10796866	0.610124	0.238571	0.670752	0.522048	0.405976
**rs6679531**	1	48524180	0.980968	0.0717496	0.255121	NA	0.125781
**rs12080303**	1	48527879	0.819063	NA	NA	NA	NA
**rs587961**	11	1881256	NA	0.581533	NA	NA	NA
**rs2137320**	11	1884342	0.0462334	0.406225	0.928157	0.404835	0.0868589
**rs4980379**	11	1888614	0.130248	0.959649	0.709274	NA	0.218798
**rs661348**	11	1905292	0.614428	NA	0.230328	NA	0.406133
**rs277129**	13	32095644	0.262344	NA	NA	NA	NA
**rs563854**	13	32101568	0.281166	0.266528	0.595277	NA	0.0854041
**rs1869799**	13	32105351	0.0730938	NA	NA	NA	NA
**rs1200470**	13	32112348	0.257875	NA	NA	NA	NA
**rs1535532**	13	32114398	0.0448605	0.132343	0.513179	0.417744	**0.00338761****
**rs1902272**	13	32125315	0.106017	NA	0.303837	0.529929	0.023535
**rs277143**	13	32132567	0.227469	NA	NA	NA	NA
**rs1671966**	13	32185002	0.0326665	0.387838	0.382306	NA	0.0145466
**rs1327981**	13	32197584	0.0150309	NA	NA	NA	NA
**rs1327980**	13	32200557	0.0149524	0.239299	0.549326	NA	**0.0059363****
**rs9603395**	13	32202333	0.0178471	NA	NA	NA	NA
**rs2025908**	13	32226399	0.0164743	0.274469	0.565943	NA	0.0081482
**rs1571303**	13	32250858	0.0164826	NA	NA	NA	NA
**rs569016**	13	70102087	0.743783	0.654834	0.698383	NA	0.85813
**rs1005002**	X	43827064	0.475073	0.544397	NA	NA	0.816715
**rs5905587**	X	43833996	0.633644	0.318775	NA	0.939525	0.915971
**rs5906426**	X	43877475	0.652005	0.300407	NA	0.6188	0.93254
**rs5906430**	X	43879037	0.813175	0.342163	NA	NA	0.479898

All SNPs listed here showed a significant or a suggestive association with the phenotype in the full discovery cohort and/or in at least one stratified analysis. Heterogeneity was tested with METASOFT as well as using the Cochran Q statistic. ** significant heterogeneity after a Bonferroni correction for the 7 independent loci identified (P<0.0072) in bold.

Supplementary Table 4. Replication of genome-wide significant/suggestive associations of SNPs with PA for men only

					Discovery cohort		German cohort		German+Italian+French#2 cohorts		
**SNP**	**chr**	**position**	**A1/A2**	**#coh**	**OR (95% CI)**	**P-value**	**OR (95% CI)**	**P-value**	**OR (FE) (95% CI)**	**P-value (FE)**	**P-value (RE)**
**rs284277**	1	10790797	C/A	3	1.8 (1.47-2.21)	1.09E-08**	1.31 (1.06-1.63)	0.0143	1.22 (1.01-1.48)	0.0443	0.0512
**rs880315**	1	10796866	C/T	4	1.69 (1.38-2.06)	3.34E-07*	1.42 (1.14-1.77)	0.00162**	1.34 (1.12-1.61)	0.00164**	0.00215**
**rs6679531**	1	48524180	C/T	3	1.1 (0.9-1.35)	0.35	1.2 (0.96-1.5)	0.108	1.32 (1.08-1.61)	0.00649	0.00763
**rs587961**	11	1881256	C/T	2	1.46 (1.19-1.79)	0.000244	1.21 (0.96-1.52)	0.106	NA	NA	NA
**rs2137320**	11	1884342	A/G	4	1.36 (1.12-1.66)	0.00237	1.24 (0.99-1.55)	0.0559	1.36 (1.13-1.63)	0.00112**	0.00148**
**rs4980379**	11	1888614	T/C	3	1.34 (1.11-1.63)	0.00298	1.3 (1.04-1.63)	0.0196	1.3 (1.07-1.58)	0.00897	0.0106
**rs661348**	11	1905292	C/T	2	1.43 (1.18-1.73)	0.000263	NA	NA	NA	NA	NA
**rs563854**	13	32101568	T/C	3	1.62 (1.34-1.96)	9.36E-07*	1.76 (1.42-2.19)	2.62E-07**	1.64 (1.35-1.96)	5.26E-07**	6.42E-07**
**rs1535532**	13	32114398	T/C	4	2.15 (1.74-2.66)	1.31E-12**	2.05 (1.62-2.6)	3.36E-09**	1.75 (1.43-2.13)	2.2E-08**	2.17E-08**
**rs1902272**	13	32125315	T/C	3	1.95 (1.56-2.43)	4.21E-09**	NA	NA	1.16 (0.81-1.67)	0.405	0.44
**rs1671966**	13	32185002	T/C	3	1.83 (1.49-2.23)	3.94E-09**	1.89 (1.51-2.37)	3.72E-08**	1.79 (1.47-2.17)	1.6E-08**	2.23E-08**
**rs1327980**	13	32200557	C/T	3	1.68 (1.39-2.03)	8.11E-08*	1.8 (1.45-2.24)	1.21E-07**	1.72 (1.41-2.08)	4.75E-08**	5.68E-08**
**rs2025908**	13	32226399	A/G	3	1.71 (1.41-2.08)	4.3E-08**	1.81 (1.45-2.25)	1.18E-07**	1.69 (1.41-2.08)	7.19E-08**	8.4E-08**
**rs569016**	13	70102087	T/C	3	0.95 (0.76-1.19)	0.641	0.78 (0.61-0.99)	0.0416	0.85 (0.68-1.05)	0.142	0.161
**rs1005002**	X	43827064	A/G	3	1.58 (1.36-1.84)	2.14E-09**	1.23 (1.05-1.45)	0.0127	1.23 (1.04-1.45)	0.0127	0.0149
**rs5905587**	X	43833996	C/T	4	1.59 (1.37-1.86)	2.31E-09**	1.16 (0.99-1.37)	0.0753	1.15 (0.98-1.33)	0.0794	0.0972
**rs5906426**	X	43877475	G/A	4	1.54 (1.33-1.8)	2.07E-08**	1.19 (1.01-1.4)	0.0433	1.19 (1.02-1.39)	0.0309	0.0387
**rs5906430**	X	43879037	T/C	2	1.53 (1.3-1.8)	3.58E-07*	1.27 (1.06-1.51)	0.00862	NA	NA	NA

All SNPs listed here showed a significant or a suggestive association with the phenotype in the full discovery cohort and/or in at least one stratified analysis.

The replication meta-analysis and the joint analysis of the discovery and replication stages were carried out as a fixed effect inverse-variance or a random-effects model meta-analysis using METASOFT. ** significant association after Bonferroni correction ($P<7.36\times10^{-8}$ for genotyped data and $P<5\times10^{-8}$ for imputed data in the discovery cohort; P< 0.0031 for the German replication cohort and the replication meta-analysis), * suggestive association in the discovery cohort ($P<10^{-6}$); A1: risk allele in the full discovery cohort; A2: second allele; #coh: number of cohorts where the SNP is available; OR (95% CI): odds ratio (95% confidence interval); FE: fixed-effect model; RE: random-effect model.

Supplementary Table 5. Replication of genome-wide significant/suggestive associations of SNPs with PA for women only

					Discovery cohort		German cohort		German+Italian+French#2 cohorts		
SNP	chr	position	A1/A2	#coh	OR (95% CI)	P-value	OR (95% CI)	P-value	OR (FE) (95% CI)	P-value (FE)	P-value (RE)
**rs284277**	1	10790797	C/A	3	1.35 (1.04-1.76)	0.0252	1.61 (1.24-2.09)	0.000414**	1.53 (1.2-1.95)	0.000543**	0.000651**
**s880315**	1	10796866	C/T	4	1.46 (1.12-1.9)	0.00527	1.61 (1.24-2.11)	0.000423**	1.51 (1.2-1.89)	0.000417**	0.000552**
**rs6679531**	1	48524180	C/T	3	2.14 (1.63-2.81)	5.25E-08**	1.07 (0.81-1.4)	0.649	0.95 (0.74-1.23)	0.715	0.746**
**rs2137320**	11	1884342	A/G	4	1.82 (1.41-2.37)	6.04E-06	1.45 (1.1-1.9)	0.00745	1.44 (1.14-1.81)	0.00201**	0.00262**
**rs4980379**	11	1888614	T/C	3	1.62 (1.26-2.09)	0.000209	1.49 (1.14-1.95)	0.00344	1.52 (1.19-1.93)	0.000664**	0.000795**
**rs661348**	11	1905292	C/T	2	1.58 (1.22-2.04)	0.000499	NA	NA	NA	NA	NA
**rs563854**	13	32101568	T/C	3	1.09 (0.85-1.4)	0.499	0.76 (0.58-0.99)	0.0407	0.74 (0.58-0.93)	0.0112	0.0132
**rs1535532**	13	32114398	T/C	4	1.13 (0.87-1.46)	0.36	0.81 (0.62-1.06)	0.119	0.78 (0.62-0.97)	0.0281	0.0353
**rs1902272**	13	32125315	T/C	3	1.18 (0.91-1.54)	0.217	NA	NA	0.8 (0.51-1.25)	0.324	0.356
**rs1671966**	13	32185002	T/C	3	1.02 (0.79-1.31)	0.898	0.98 (0.75-1.29)	0.909	0.92 (0.72-1.16)	0.463	0.498
**rs1327980**	13	32200557	C/T	3	1.05 (0.82-1.34)	0.721	0.77 (0.59-1.01)	0.0611	0.77 (0.6-0.98)	0.0362	0.042
**rs2025908**	13	32226399	A/G	3	1.03 (0.8-1.32)	0.801	0.8 (0.61-1.05)	0.106	0.84 (0.66-1.06)	0.145	0.163
**rs569016**	13	70102087	T/C	3	2.21 (1.61-3.02)	7.38E-07*	0.9 (0.67-1.21)	0.484	0.95 (0.72-1.23)	0.699	0.731
**rs1005002**	X	43827064	A/G	2	1.56 (1.18-2.06)	0.00158	1.03 (0.79-1.36)	0.809	NA	NA	NA
**rs5905587**	X	43833996	C/T	3	1.67 (1.26-2.21)	0.000382	1.05 (0.8-1.39)	0.729	1.14 (0.87-1.47)	0.36	0.393
**rs5906426**	X	43877475	G/A	3	1.65 (1.25-2.19)	0.000459	1.02 (0.77-1.34)	0.891	1.1 (0.85-1.43)	0.456	0.492
**rs5906430**	X	43879037	T/C	2	1.67 (1.24-2.25)	0.000804	0.95 (0.71-1.26)	0.711	NA	NA	NA

All SNPs listed here showed a significant or a suggestive association with the phenotype in the full discovery cohort and/or in at least one stratified analysis.

The replication meta-analysis and the joint analysis of the discovery and replication stages were carried out as a fixed effect inverse-variance or a random-effects model meta-analysis using METASOFT. ** significant association after Bonferroni correction ($P<7.36\times10^{-8}$ for genotyped data and $P<5\times10^{-8}$ for imputed data in the discovery cohort; P< 0.0031 for the German replication cohort and the replication meta-analysis), * suggestive association in the discovery cohort ($P<10^{-6}$); A1: risk allele in the full discovery cohort; A2: second allele; #coh: number of cohorts where the SNP is available; OR (95% CI): odds ratio (95% confidence interval); FE: fixed-effect model; RE: random-effect model.

Supplementary Table 6. Replication of genome-wide significant/suggestive associations of SNPs with APA

					Discovery cohort		German cohort		German+Italian+French#2 cohorts		
**SNP**	**chr**	**position**	**A1/A2**	**#coh**	**OR (95% CI)**	**P-value**	**OR (95% CI)**	**P-value**	**OR (FE) (95% CI)**	**P-value (FE)**	**P-value (RE)**
**rs284277**	1	10790797	C/A	3	1.6 (1.33-1.94)	1.22E-06	1.48 (1.2-1.83)	0.000319**	1.37 (1.14-1.65)	0.000914**	0.00109**
**rs880315**	1	10796866	C/T	4	1.63 (1.35-1.98)	5.46E-07*	1.51 (1.22-1.88)	0.000147**	1.4 (1.18-1.67)	0.000143**	0.000191**
**rs6679531**	1	48524180	C/T	3	1.41 (1.16-1.71)	0.000613	1.09 (0.87-1.36)	0.437	1.09 (0.9-1.32)	0.378	0.412
**rs587961**	11	1881256	C/T	2	1.67 (1.36-2.05)	1.04E-06	1.16 (0.93-1.46)	0.193	NA	NA	NA
**rs2137320**	11	1884342	A/G	4	1.74 (1.44-2.11)	1.18E-08**	1.36 (1.09-1.69)	0.00615	1.42 (1.19-1.69)	8.97E-05**	0.00012**
**rs4980379**	11	1888614	T/C	3	1.6 (1.33-1.93)	8.78E-07*	1.34 (1.07-1.66)	0.0093	1.37 (1.14-1.66)	0.00104**	0.00125**
**rs661348**	11	1905292	C/T	2	1.53 (1.27-1.84)	6.67E-06	NA	NA	NA	NA	NA
**rs563854**	13	32101568	T/C	3	1.29 (1.08-1.55)	0.00549	1.29 (1.04-1.59)	0.019	1.19 (1-1.43)	0.0544	0.0627
**rs1535532**	13	32114398	T/C	4	1.46 (1.21-1.77)	0.000107	1.37 (1.09-1.71)	0.00613	1.2 (1-1.43)	0.047	0.0535
**rs1902272**	13	32125315	T/C	3	1.41 (1.16-1.72)	0.000717	NA	NA	1.06 (0.78-1.44)	0.706	0.738
**rs1671966**	13	32185002	T/C	3	1.27 (1.06-1.54)	0.011	1.5 (1.2-1.87)	0.000352**	1.33 (1.11-1.61)	0.00251**	0.00265**
**rs1327980**	13	32200557	C/T	3	1.22 (1.02-1.46)	0.0317	1.3 (1.05-1.62)	0.0163	1.24 (1.03-1.5)	0.021	0.0245
**rs2025908**	13	32226399	A/G	3	1.23 (1.02-1.47)	0.0274	1.32 (1.06-1.63)	0.0133	1.27 (1.06-1.53)	0.0107	0.0126
**rs569016**	13	70102087	T/C	3	1.26 (1.01-1.56)	0.0372	0.84 (0.66-1.07)	0.152	1.12 (0.91-1.38)	0.294	0.324
**rs1005002**	X	43827064	A/G	2	1.51 (1.28-1.77)	7.17E-07*	1.27 (1.06-1.53)	0.0108	NA	NA	NA
**rs5905587**	X	43833996	C/T	3	1.56 (1.32-1.84)	1.89E-07*	1.25 (1.04-1.51)	0.0192	1.23 (1.04-1.47)	0.0153	0.0179
**rs5906426**	X	43877475	G/A	3	1.52 (1.29-1.79)	8.02E-07*	1.26 (1.04-1.52)	0.0158	1.25 (1.05-1.49)	0.00989	0.0116
**rs5906430**	X	43879037	T/C	2	1.56 (1.31-1.87)	1.23E-06	1.29 (1.06-1.57)	0.0114	NA	NA	NA

All SNPs listed here showed a significant or a suggestive association with the phenotype in the full discovery cohort and/or in at least one stratified analysis. The replication meta-analysis and the joint analysis of the discovery and replication stages were carried out as a fixed effect inverse-variance or a random-effects model meta-analysis using METASOFT. ** significant association after Bonferroni correction ($P<7.36\times10^{-8}$ for genotyped data and $P<5\times10^{-8}$ for imputed data in the discovery cohort; P< 0.0031 for the German replication cohort and the replication meta-analysis) , * suggestive association in the discovery cohort ($P<10^{-6}$); A1: risk allele in the full discovery cohort; A2: second allele; #coh: number of cohorts where the SNP is available; OR (95% CI): odds ratio (95% confidence interval); FE: fixed-effect model; RE: random-effect model.

Supplementary Table 7. Replication of genome-wide significant/suggestive associations of SNPs with BAH

					Discovery cohort		German cohort		German+Italian+French#2 cohorts		
SNP	chr	position	A1/A2	#coh	OR (95% CI)	P-value	OR (95% CI)	P-value	OR (FE) (95% CI)	P-value (FE)	P-value (RE)
**rs284277**	1	10790797	C/A	3	1.61 (1.3-1.99)	1.28E-05	1.19 (0.91-1.55)	0.215	1.12 (0.88-1.43)	0.358	0.391
**rs880315**	1	10796866	C/T	4	1.52 (1.23-1.87)	0.000117	1.23 (0.94-1.61)	0.134	1.23 (0.97-1.55)	0.0826	0.101
**rs6679531**	1	48524180	C/T	3	1.41 (1.14-1.75)	0.00188	1.5 (1.15-1.97)	0.0029**	1.53 (1.2-1.94)	0.00064**	0.000766**
**rs2137320**	11	1884342	A/G	4	1.3 (1.05-1.61)	0.0159	1.17 (0.89-1.54)	0.271	1.3 (1.03-1.65)	0.0252	0.0317
**rs4980379**	11	1888614	T/C	3	1.29 (1.04-1.59)	0.0196	1.32 (1.01-1.74)	0.0438	1.32 (1.03-1.69)	0.0265	0.0309
**rs661348**	11	1905292	C/T	2	1.42 (1.16-1.75)	0.000817	NA	NA	NA	NA	NA
**rs563854**	13	32101568	T/C	3	1.5 (1.22-1.85)	0.000116	1.56 (1.2-2.04)	0.00104**	1.45 (1.149-1.85)	0.00167**	0.00199**
**rs1535532**	13	32114398	T/C	4	1.98 (1.58-2.49)	3.89E-09**	1.82 (1.36-2.44)	6.71E-05**	1.61 (1.27-2.08)	0.000121**	0.000162**
**rs1902272**	13	32125315	T/C	3	1.82 (1.44-2.31)	7.77E-07*	NA	NA	1.33 (0.82-2.13)	0.249	0.276
**rs1671966**	13	32185002	T/C	3	1.74 (1.4-2.16)	5.81E-07*	1.76 (1.32-2.33)	0.000104**	1.64 (1.28-2.13)	9.69E-05**	0.000117**
**rs1327980**	13	32200557	C/T	3	1.72 (1.39-2.11)	3.55E-07*	1.6 (1.22-2.1)	0.000595**	1.54 (1.21-1.96)	0.000402**	0.000482**
**rs2025908**	13	32226399	A/G	3	1.72 (1.4-2.13)	3.64E-07*	1.59 (1.22-2.08)	0.000663**	1.55 (1.22-1.97)	0.000305**	0.000366**
**rs569016**	13	70102087	T/C	3	1.33 (1.04-1.7)	0.0232	0.77 (0.58-1.03)	0.0818	0.84 (0.65-1.1)	0.201	0.224
**rs1005002**	X	43827064	A/G	2	1.65 (1.38-1.98)	5.99E-08**	1.16 (0.94-1.45)	0.173	NA	NA	NA
**rs5905587**	X	43833996	C/T	3	1.66 (1.38-1.99)	8.69E-08*	1.08 (0.87-1.35)	0.493	1.09 (0.89-1.35)	0.401	0.435
**rs5906426**	X	43877475	G/A	3	1.61 (1.34-1.93)	3.65E-07*	1.08 (0.87-1.35)	0.484	1.11 (0.91-1.37)	0.296	0.326
**rs5906430**	X	43879037	T/C	2	1.52 (1.25-1.84)	2.5E-05	1.11 (0.89-1.4)	0.355	NA	NA	NA

All SNPs listed here showed a significant or a suggestive association with the phenotype in the full discovery cohort and/or in at least one stratified analysis.

The replication meta-analysis and the joint analysis of the discovery and replication stages were carried out as a fixed effect inverse-variance or a random-effects model meta-analysis using METASOFT. **significant association after Bonferroni correction ($P<7.36\times10^{-8}$ for genotyped data and $P<5\times10^{-8}$ for imputed data in the discovery cohort; P< 0.0031 for the German replication cohort and the replication meta-analysis) , *suggestive association in the discovery cohort ($P<10^{-6}$); A1: risk allele in the full discovery cohort; A2: second allele; #coh: number of cohorts where the SNP is available; OR (95% CI): odds ratio (95% confidence interval); FE: fixed-effect model; RE: random-effect model.

Supplementary Table 8. Replication in the Italian cohort of genome-wide significant/suggestive associations of SNPs with PA

				All		Men		Women		APA		BAH	
**SNP**	**CHR**	**BP**	**A1**	**OR**	**P-value**	**OR**	**P-value**	**OR**	**P-value**	**OR**	**P-value**	**OR**	**P-value**
**rs284277**	1	10790797	C	1 (0,71-1,42)	0,984	0,91 (0,58-1,41)	0,663	1,19 (0,66-2,15)	0,57	1,07 (0,73-1,58)	0,734	0,86 (0,47-1,55)	0,608
**rs880315**	1	10796866	C	1,1 (0,78-1,54)	0,586	1,1 (0,72-1,69)	0,664	1,04 (0,58-1,86)	0,908	1,14 (0,78-1,67)	0,497	0,98 (0,55-1,76)	0,949
**rs6679531**	1	48524180	C	1,22 (0,87-1,72)	0,246	1,95 (1,24-3,07)	0,003	0,55 (0,3-1,02)	0,056	1,09 (0,73-1,62)	0,678	1,62 (0,92-2,85)	0,092
**rs2137320**	11	1884342	A	1,5 (1,09-2,08)	0,013	1,58 (1,05-2,39)	0,028	1,36 (0,78-2,37)	0,282	1,53 (1,06-2,21)	0,022	1,49 (0,87-2,54)	0,146
**rs4980379**	11	1888614	T	1,42 (1,02-1,98)	0,038	1,29 (0,84-1,98)	0,24	1,64 (0,95-2,84)	0,077	1,48 (1,02-2,15)	0,041	1,3 (0,75-2,27)	0,354
**rs661348**	11	1905292	C	1,27 (0,92-1,75)	0,14	1,18 (0,78-1,79)	0,424	1,47 (0,87-2,49)	0,149	1,44 (1-2,07)	0,052	0,96 (0,56-1,66)	0,884
**rs563854**	13	32101568	T	1,02 (0,75-1,39)	0,906	1,23 (0,82-1,84)	0,309	0,65 (0,38-1,11)	0,114	0,97 (0,68-1,38)	0,858	1,14 (0,69-1,89)	0,6
**rs1535532**	13	32114398	T	0,99 (0,72-1,37)	0,955	1,31 (0,85-2,02)	0,222	0,61 (0,36-1,03)	0,065	0,93 (0,64-1,36)	0,724	1,16 (0,69-1,96)	0,582
**rs1902272**	13	32125315	T	0,98 (0,69-1,38)	0,905	1,11 (0,71-1,76)	0,643	0,74 (0,43-1,3)	0,296	0,89 (0,6-1,31)	0,557	1,29 (0,72-2,3)	0,399
**rs1671966**	13	32185002	T	1,07 (0,77-1,49)	0,698	1,46 (0,93-2,28)	0,1	0,69 (0,41-1,17)	0,168	0,98 (0,67-1,43)	0,921	1,32 (0,77-2,26)	0,321
**rs1327980**	13	32200557	C	1,16 (0,84-1,59)	0,38	1,43 (0,94-2,16)	0,096	0,77 (0,45-1,33)	0,348	1,09 (0,76-1,57)	0,637	1,33 (0,78-2,25)	0,291
**rs2025908**	13	32226399	A	1,23 (0,89-1,69)	0,202	1,38 (0,91-2,09)	0,134	0,99 (0,58-1,67)	0,96	1,17 (0,81-1,67)	0,404	1,4 (0,84-2,36)	0,201
**rs569016**	13	70102087	T	1,16 (0,8-1,68)	0,44	1,21 (0,75-1,95)	0,443	1,22 (0,64-2,34)	0,546	1,09 (0,71-1,67)	0,683	1,27 (0,67-2,42)	0,463
**rs1005002**	X	43827064	A	NA	NA	11980 (0-9,264E+127)	0,948	NA	NA	NA	NA	NA	NA
**rs5905587**	X	43833996	C	NA	NA	0 (0-2,258E+186)	0,968	NA	NA	NA	NA	NA	NA
**rs5906426**	X	43877475	G	NA	NA	0 (0-2,258E+186)	0,968	NA	NA	NA	NA	NA	NA

All SNPs listed here showed a significant or a suggestive association with the phenotype in the full discovery cohort and/or in at least one stratified analysis. Associations were tested using a logistic regression model in PLINK 1.9. **significant association after Bonferroni correction for the 16 SNPs tested (P<0.0031); A1: risk allele in the full discovery cohort; OR (95% CI): odds ratio (95% confidence interval).

Supplementary Table 9. Replication in the second French cohort of genome-wide significant/suggestive associations of SNPs with PA

				All		Men		Women		APA		BAH	
**SNP**	**CHR**	**BP**	**A1**	**OR**	**P-value**	**OR**	**P-value**	**OR**	**P-value**	**OR**	**P-value**	**OR**	**P-value**
**rs880315**	1	10719453	C	1,44 (0,93-2,21)	0,099	1,32 (0,74-2,35)	0,347	1,62 (0,82-3,19)	0,167	1,33 (0,81-2,2)	0,263	1,79 (0,84-3,79)	0,129
**rs2137320**	11	1840918	A	1,72 (1,13-2,64)	0,012	1,84 (1,04-3,26)	0,035	1,5 (0,74-3,02)	0,258	1,53 (0,95-2,47)	0,081	2,22 (1,07-4,61)	0,033
**rs1535532**	13	31012398	T	1,11 (0,72-1,71)	0,629	1,17 (0,67-2,04)	0,585	0,9 (0,44-1,84)	0,779	0,99 (0,61-1,58)	0,954	1,46 (0,64-3,31)	0,367
**rs1902272**	13	31023315	T	1,13 (0,72-1,78)	0,6	1,26 (0,7-2,26)	0,447	0,91 (0,43-1,91)	0,801	1,04 (0,63-1,7)	0,89	1,42 (0,61-3,33)	0,419
**rs5905587**	X	43718940	C	1,21 (0,85-1,73)	0,297	1,06 (0,71-1,59)	0,779	2,15 (0,95-4,89)	0,067	1,16 (0,77-1,74)	0,475	1,19 (0,65-2,18)	0,567
**rs5906426**	X	43762419	G	1,32 (0,92-1,9)	0,128	1,17 (0,78-1,77)	0,446	2,22 (0,98-5,02)	0,055	1,21 (0,81-1,81)	0,354	1,47 (0,78-2,77)	0,239

All SNPs listed here showed a significant or a suggestive association with the phenotype in the full discovery cohort and/or in at least one stratified analysis.

Associations were tested using a logistic regression model in PLINK 1.9. **significant association after Bonferroni correction for the 6 SNPs tested (P< 0.0083); A1: risk allele in the full discovery cohort; OR (95% CI): odds ratio (95% confidence interval).

Supplementary Table 10. Global meta-analysis of genome-wide significant/suggestive associations of SNPs with PA for men and women

				Men				Women			
SNP	chr	position	A1/A2	#coh	OR (FE) (95% CI)	P-value (FE)	P-value (RE)	#coh	OR (FE) (95% CI)	P-value (FE)	P-value (RE)
**rs284277**	1	10790797	C/A	3	1.47 (1.28-1.69)	6.09E-08**	9.45E-09**	3	1.45 (1.21-1.73)	4.84E-05	7.24E-05
**rs880315**	1	10796866	C/T	4	1.49 (1.3-1.71)	8.06E-09**	1.70E-08**	4	1.49 (1.25-1.77)	6.96E-06	1.16E-05
**rs6679531**	1	48524180	C/T	3	1.21 (1.05-1.4)	0.0092	0.0128	3	1.38 (1.15-1.66)	0.000654	5.86E-09**
**rs587961**	11	1881256	C/T	2	0.74 (0.64-0.87)	0.000136	0.000194	-	-	-	-
**rs2137320**	11	1884342	A/G	4	1.36 (1.19-1.55)	8.39E-06	1.53E-05	4	1.6 (1.34-1.9)	1.09E-07	1.89E-07
**rs4980379**	11	1888614	T/C	3	1.32 (1.15-1.52)	7.85E-05	0.000126	3	1.57 (1.31-1.87)	5.15E-07	8.34E-07
**rs661348**	11	1905292	C/T	2	1.38 (1.16-1.65)	0.000267	0.000374	2	1.56 (1.24-1.96)	0.000169	0.000223
**rs563854**	13	32101568	T/C	3	1.62 (1.42-1.86)	2.29E-12**	6.55E-12**	3	1.13 (0.95-1.34)	0.173	0.0931
**rs1535532**	13	32114398	T/C	4	1.92 (1.67-2.22)	4.55E-19**	1.94E-18**	4	1.1 (0.92-1.3)	0.292	0.202
**rs1902272**	13	32125315	T/C	3	1.69 (1.4-2.04)	5.34E-08**	3.85E-08**	3	0.94 (0.75-1.18)	0.574	0.629
**rs1671966**	13	32185002	T/C	3	1.81 (1.57-2.09)	3.38E-16**	1.33E-15**	3	1.04 (0.87-1.24)	0.656	0.706
**rs1327980**	13	32200557	C/T	3	1.7 (1.48-1.94)	1.96E-14**	6.69E-14**	3	0.89 (0.75-1.06)	0.209	0.234
**rs2025908**	13	32226399	A/G	3	1.71 (1.49-1.96)	1.56E-14**	5.37E-14**	3	0.93 (0.78-1.1)	0.388	0.442
**rs569016**	13	70102087	T/C	3	0.9 (0.77-1.05)	0.168	0.205	3	1.36 (1.11-1.66)	0.00342	3.61E-07
**rs1005002**	X	43827064	A/G	3	1.41 (1.26-1.58)	1.07E-09**	8.35E-10**	2	1.27 (1.04-1.54)	0.0165	0.00902
**rs5905587**	X	43833996	C/T	4	1.35 (1.21-1.51)	4.66E-08**	4.01E-09**	3	1.35 (1.12-1.64)	0.00201	0.000674
**rs5906426**	X	43877475	G/A	4	1.35 (1.22-1.51)	3.78E-08**	2.05E-08**	3	1.33 (1.1-1.61)	0.00337	0.000727
**rs5906430**	X	43879037	T/C	2	1.4 (1.24-1.58)	3.49E-08**	5.69E-08**	2	1.24 (1.01-1.52)	0.0418	0.00309

All SNPs listed here showed a significant or a suggestive association with the phenotype in the full discovery cohort and/or in at least one stratified analysis.

The global meta-analysis was carried out as a fixed effect inverse-variance or a random-effects model meta-analysis using METASOFT. ** significant association after Bonferroni correction ($P<7.36 \times 10^{-8}$); A1: risk allele in the full discovery cohort; A2: second allele; #studies: number of cohorts where the SNP is available; OR (95% CI): odds ratio (95% confidence interval); FE: fixed-effect model; RE: random-effect model.

Supplementary Table 11. Global meta-analysis of genome-wide significant/suggestive associations of SNPs with APA and BAH

						APA				BAH	
**SNP**	**chr**	**position**	**A1/A2**	**#studies**	**OR (FE) (95% CI)**	**P-value (FE)**	**P-value (RE)**	**#studies**	**OR (FE) (95% CI)**	**P-value (FE)**	**P-value (RE)**
**rs284277**	1	10790797	C/A	3	1.48 (1.29-1.69)	8.39E-09**	1.55E-08**	3	1.38 (1.17-1.62)	9.97E-05	0.000106
**rs880315**	1	10796866	C/T	4	1.5 (1.32-1.71)	6.22E-10**	1.33E-09**	4	1.38 (1.18-1.61)	5.84E-05	0.000115
**rs6679531**	1	48524180	C/T	3	1.24 (1.08-1.42)	0.00236	0.00301	3	1.46 (1.24-1.72)	4.36E-06	8.84E-06
**rs587961**	11	1881256	C/T	2	1.42 (1.22-1.65)	7.11E-06	2.63E-06	-	-	-	-
**rs2137320**	11	1884342	A/G	4	1.56 (1.37-1.77)	1.59E-11**	3.81E-11**	4	1.3 (1.11-1.53)	0.001	0.00174
**rs4980379**	11	1888614	T/C	3	1.48 (1.3-1.69)	6.64E-09**	1.25E-08**	3	1.3 (1.11-1.53)	0.0013	0.00209
**rs661348**	11	1905292	C/T	2	1.51 (1.28-1.78)	9.80E-07	1.48E-06	2	1.35 (1.12-1.64)	0.00208	0.00298
**rs563854**	13	32101568	T/C	3	1.24 (1.09-1.41)	0.000885	0.00129	3	1.48 (1.27-1.73)	6.72E-07	1.47E-06
**rs1535532**	13	32114398	T/C	4	1.31 (1.15-1.5)	4.25E-05	7.09E-05	4	1.8 (1.53-2.13)	4.18E-12**	1.73E-11**
**rs1902272**	13	32125315	T/C	3	1.25 (1.06-1.48)	0.00848	0.00663	3	1.71 (1.38-2.12)	7.80E-07	1.70E-06
**rs1671966**	13	32185002	T/C	3	1.31 (1.14-1.49)	8.44E-05	0.000129	3	1.7 (1.44-2.01)	2.45E-10**	7.85E-10**
**rs1327980**	13	32200557	C/T	3	1.23 (1.08-1.4)	0.00164	0.00234	3	1.64 (1.4-1.92)	7.08E-10**	2.13E-09**
**rs2025908**	13	32226399	A/G	3	1.25 (1.1-1.42)	0.000777	0.00113	3	1.65 (1.41-1.93)	5.48E-10**	1.68E-09**
**rs569016**	13	70102087	T/C	3	1.05 (0.91-1.22)	0.494	0.0914	3	1.08 (0.9-1.29)	0.424	0.023
**rs1005002**	X	43827064	A/G	2	1.4 (1.24-1.58)	6.40E-08**	9.65E-08	2	1.43 (1.25-1.65)	4.49E-07	1.91E-07
**rs5905587**	X	43833996	C/T	3	1.39 (1.23-1.57)	6.09E-08**	8.75E-08	3	1.38 (1.2-1.58)	5.40E-06	4.22E-07
**rs5906426**	X	43877475	G/A	3	1.38 (1.23-1.56)	9.74E-08	1.73E-07	3	1.37 (1.19-1.57)	6.70E-06	1.20E-06
**rs5906430**	X	43879037	T/C	2	1.43 (1.25-1.64)	1.27E-07	1.95E-07	2	1.33 (1.15-1.54)	0.000136	0.000119

All SNPs listed here showed a significant or a suggestive association with the phenotype in the full discovery cohort and/or in at least one stratified analysis.

The global meta-analysis was carried out as a fixed effect inverse-variance or a random-effects model meta-analysis using METASOFT. ** significant association after Bonferroni correction ($P<7.36 \times 10^{-8}$); A1: risk allele in the full discovery cohort; A2: second allele; #studies: number of cohorts where the SNP is available; OR (95% CI): odds ratio (95% confidence interval); FE: fixed-effect model; RE: random-effect model.

Supplementary Table 12. Adrenal eQTLs in loci reaching genome-wide and suggestive significance in the discovery cohort

SNP	chr	position	Gene	Alleles	NES	P-Value
rs277129	13	32095644	RXFP2	C_A	-0.64	7.4e-12
**rs563854**	13	32101568	RXFP2	T_C	-0.55	3.5e-10
rs1869799	13	32105351	RXFP2	T_C	-0.57	2.0e-10
rs1200470	13	32112348	RXFP2	T_C	-0.54	2.1e-10
**rs1535532**	13	32114398	RXFP2	T_C	-0.57	7.9e-11
**rs1902272**	13	32125315	RXFP2	T_C	-0.47	3.7e-7
rs277143	13	32132567	RXFP2	G_A	-0.56	1.3e-10
**rs1671966**	13	32185002	RXFP2	T_C	-0.58	1.5e-11
rs1327981	13	32197584	RXFP2	T_G	0.54	2.7e-11
**rs1327980**	13	32200557	RXFP2	T_C	0.57	8.6e-12
rs9603395	13	32202333	RXFP2	C_A	-0.47	4.9e-7
**rs2025908**	13	32226399	RXFP2	G_A	0.58	8.7e-12
rs1571303	13	32250858	RXFP2	C_T	0.57	1.4e-11
**rs1005002**	X	43827064	NDP	G_A	-0.29	0.000013
**rs5905587**	X	43833996	NDP	T_C	-0.30	0.0000050

NES, normalized effect size according to GTEX (https://gtexportal.org). The direction of effect refers to the second allele indicated in the table. Position: genome build 37. SNPs indicated in italic were not available for meta-analysis.

Supplementary Table 13. Publicly available ATAC-seq and ChIP-seq datasets used in this study, accessible through the ENCODE portal (https://www.encodeproject.org/)

Public ressources on Homo sapiens adrenal gland tissue	Laboratory	Bigwig	Identifier
ATAC-seq			
male adult (37 years)	M.Snyder	ENCFF530IBY	https://www.encodeproject.org/experiments/ENCSR548KIL/
male adult (26 years)	M.Snyder	ENCFF188RJO	https://www.encodeproject.org/experiments/ENCSR542RNG/
female adult (47 years)	M.Snyder	ENCFF593HQT	https://www.encodeproject.org/experiments/ENCSR651SOJ/
female adult (59 years)	M.Snyder	ENCFF888OID	https://www.encodeproject.org/experiments/ENCSR241OBO/
ChIP-seq			
H3K4me3 - female adult (41 years)	B. Bernstein	ENCFF243VIK	https://www.encodeproject.org/experiments/ENCSR775NSL/
H3K4me3 - male adult (34 years)	B. Ren	ENCFF389SDP	https://www.encodeproject.org/experiments/ENCSR234YIU/
H3K27ac - female adult (41 years)	B. Bernstein	ENCFF247UAC	https://www.encodeproject.org/experiments/ENCSR837CSL/
H3K27ac - male adult (34 years)	B. Ren	ENCFF788SJX	https://www.encodeproject.org/experiments/ENCSR181JFC/
H3K4me1 - female adult (41 years)	B. Bernstein	ENCFF392JRV	https://www.encodeproject.org/experiments/ENCSR038KST/
H3K4me1 - male adult (54 years)	B. Bernstein	ENCFF888LDS	https://www.ncbi.nlm.nih.gov/geo/query/acc.cgi?acc=GSM2534000
CTCF - female adult (41 years)	B. Bernstein	ENCFF089TYQ	https://www.encodeproject.org/experiments/ENCSR188XCX/

Supplementary Table 14. Primers for reference genes used for real-time RT-qPCR

Gene Symbol	Forward primer	Reverse primer
18S	CCCTGCCTTTGTACACACC	CGATCCGAGGGCCTCACTA
HPRT	CTCAACTTTAACTGGAAAGAATGTC	TCCTTTTCACCAGCAAGCT
GAPDH	TGCACCACCAACTGCTTAGC	GGCATGGACTGTGGTCATGAG

Supplementary References

1. Zhou, J. et al. Transcriptome Pathway Analysis of Pathological and Physiological Aldosterone-Producing Human Tissues. Hypertension 68, 1424-1431 (2016).
2. Nishimoto, K. et al. Aldosterone-stimulating somatic gene mutations are common in normal adrenal glands. Proc Natl Acad Sci U S A 112, E4591-9 (2015).
3. Pignatti, E., Leng, S., Carlone, D.L. & Breault, D.T. Regulation of zonation and homeostasis in the adrenal cortex. Mol Cell Endocrinol 441, 146-155 (2017).
4. Del Valle, I. *et al.* A genomic atlas of human adrenal and gonad development. *Wellcome Open Res* **2**, 25 (2017).
