# Supplementary material for: Identification of risk loci for primary aldosteronism in genome-wide association studies
Source: Nat Commun. 2022 Sep 3;13:5198. doi: 10.1038/s41467-022-32896-8 (PMC9440917; doi:10.1038/s41467-022-32896-8)
Supplement: Supplementary file 2 — Description of Additional Supplementary Files [file 41467_2022_32896_MOESM2_ESM.pdf]

Description of Additional Supplementary Files

File Name: Supplementary Data 1
Description**: Complete list of genome-wide significant/suggestive associations of SNPs with PA in the discovery cohort.** All SNPs listed here showed a significant or a suggestive association with the phenotype in the full discovery cohort and/or in at least one stratified analysis. Associations were tested using a logistic regression model in PLINK 1.9. ** significant association after Bonferroni correction (P<7.36 ×10-8) in bold, * suggestive association (P<10-6); position: genome build 37; A1 : risk allele; A2: second allele; Freq: risk allele frequency; OR (CI): odds ratio (95% confidence interval).

File Name: Supplementary Data 2
Description: **Genome-wide significant associations of SNPs with PA in the discovery cohort after imputation.** Results for the entire discovery cohort and subanalyses for men, women, APA and BAH are presented in the different sheets. Conditional analysis of the locus on chr11 is presented in the sheet conditional_analysis_chr11). Variants with a significant association after Bonferroni correction (P<5 ×10-8) with the phenotype are presented. Position: genome build 37; A1 : risk allele; A2: second allele; OR: odds ratio; L95, U95: lower and upper 95% confidence interval. Associations and conditional analysis were performed using a logistic regression implemented in PLINK version 2.0.2.3.

File Name: Supplementary Data 3
Description: **Replication of genome-wide significant/suggestive associations of SNPs with PA and meta-analysis.** Complete list of SNPs showing a significant or a suggestive association with the phenotype in the full discovery cohort and/or in at least one stratified analysis. The replication meta-analysis and the joint analysis of the discovery and replication stages were carried out as a fixed effect inverse-variance or a random-effects model meta-analysis using METASOFT. ** significant association after Bonferroni correction (P<7.36 ×10-8 for genotyped data and P<5×10-8 for imputed data in the discovery cohort; P<7.36 ×10-8 for the global meta-analysis; P< 0.0031 for the German replication cohort and for the replication meta-analysis of the German, Italian and second French cohorts), * suggestive association in the discovery cohort (P<10-6). A1: risk allele in the full discovery cohort; A2: second allele; #coh: number of cohorts where the SNP is available; OR (95% CI): odds ratio (95% confidence interval); FE: fixed-effect model; RE: random-effect model.

File Name: Supplementary Data 4
Description**: Adrenal eQTLs in loci reaching genome-wide significance in the discovery cohort after imputation.** NES, normalized effect size according to GTEX (https://gtexportal.org). The direction of effect refers to the second allele indicated the column Alleles. A1: risk allele in the full discovery cohort; A2: second allele. Position: genome build 37.
