# Supplementary material for: Identification of risk loci for primary aldosteronism in genome-wide association studies
Source: Nat Commun. 2022 Sep 3;13:5198. doi: 10.1038/s41467-022-32896-8 (PMC9440917; doi:10.1038/s41467-022-32896-8)
Supplement: Supplementary file 7 — Reporting Summary [file 41467_2022_32896_MOESM7_ESM.pdf]

Corresponding author(s): Maria-Christina Zennaro
Last updated by author(s): Jul 20, 2022

Reporting Summary

Nature Portfolio wishes to improve the reproducibility of the work that we publish. This form provides structure for consistency and transparency in reporting. For further information on Nature Portfolio policies, see our Editorial Policies and the Editorial Policy Checklist.

Statistics

For all statistical analyses, confirm that the following items are present in the figure legend, table legend, main text, or Methods section.

n/a	Confirmed	
☐	☒	The exact sample size (*n*) for each experimental group/condition, given as a discrete number and unit of measurement
☐	☒	A statement on whether measurements were taken from distinct samples or whether the same sample was measured repeatedly
☐	☒	The statistical test(s) used AND whether they are one- or two-sided<br>*Only common tests should be described solely by name; describe more complex techniques in the Methods section.*
☐	☒	A description of all covariates tested
☐	☒	A description of any assumptions or corrections, such as tests of normality and adjustment for multiple comparisons
☐	☒	A full description of the statistical parameters including central tendency (e.g. means) or other basic estimates (e.g. regression coefficient) AND variation (e.g. standard deviation) or associated estimates of uncertainty (e.g. confidence intervals)
☐	☒	For null hypothesis testing, the test statistic (e.g. *F*, *t*, *r*) with confidence intervals, effect sizes, degrees of freedom and *P* value noted<br>*Give P values as exact values whenever suitable.*
☒	☐	For Bayesian analysis, information on the choice of priors and Markov chain Monte Carlo settings
☐	☒	For hierarchical and complex designs, identification of the appropriate level for tests and full reporting of outcomes
☐	☒	Estimates of effect sizes (e.g. Cohen's *d*, Pearson's *r*), indicating how they were calculated

Our web collection on statistics for biologists contains articles on many of the points above.

Software and code

Policy information about availability of computer code

Data collection	No software was used to collect the data.
Data analysis	Primary analysis of the GWAS results was done using the GenomeStudio software (Illumina®, San Diego, USA).<br>PLINK version 1.9 (for genotype data) and version 2.0.2.3 (for imputed dosage data) was used to test the association with primary aldosteronism.<br>METASOFT version 2.0.0 was used for meta-analysis ans heterogeneity analysis.<br>SHAPEIT version 2 (r837) (Delaneau et al, Nat Methods. 9(2):179-81, 2012) was used to phase SNPs in common between cases and controls in the discovery cohort.<br>Minimac4 version 4/1.0.0 program (Das et al, Nat Genet. Oct;48(10):1284-1287, 2016) was used for the imputation of SNPs and indels<br>pyGenometracks package was used to visualize ENCODE ATAC-seq and ChIP-seq files from adrenal gland.<br>UCSC lift-over online tool (https://genome.ucsc.edu/cgi-bin/hgLiftOver) was used to convert original assemblies used in GWAS into GRCh38 using chromosome coordinates.<br>BD FACSAria III and the BD FACSDIVA software were used to FACS sort nuclei and to exclude debris.<br>Cell Ranger Single-Cell Software Suite 3.0.2 was used in single nucleus RNA sequencing analysis.<br>Visualizations, clustering and differential expression tests of single nucleus RNA sequencing were performed in R (v 3.4.3) using Seurat (v3.0.2).<br>Multi gauge software (Fujifilm, Tokyo-Japan) was used for Western Blot quantification.<br>Graphpad Prism 9 (GraphPad software Inc, San Diego, CA) or MedCalc19 (MedCalc software Ltd) were used for cell experiments statistics.

For manuscripts utilizing custom algorithms or software that are central to the research but not yet described in published literature, software must be made available to editors and reviewers. We strongly encourage code deposition in a community repository (e.g. GitHub). See the Nature Portfolio guidelines for submitting code & software for further information.

Data

Policy information about availability of data

All manuscripts must include a data availability statement. This statement should provide the following information, where applicable:

- Accession codes, unique identifiers, or web links for publicly available datasets
- A description of any restrictions on data availability
- For clinical datasets or third party data, please ensure that the statement adheres to our policy

Publicly available ATAC-seq and ChIP-seq datasets used in this study were accessible through the ENCODE portal (https://www.encodeproject.org/)
Genotype-Tissue Expression (GTEx, v8) was queried for eQTL analysis with rsID of variants at PA loci on 20220422
GWAS catalog database was queried on 20211210
Summary statistics of the discovery cohort that support the findings of this GWAS has been deposited in the GWAS catalogue database under accession codes GCST90129615, GCST90129616, GCST90129617, GCST90129618, GCST90129619, GCST90129620, GCST90129621, GCST90129622, GCST90129623, GCST90129624 [[https://www.ebi.ac.uk/gwas/]. The individual level genotype data will not be publicly available since they contain information that could compromise research participant privacy and consent. snRNAseq data have been deposited in the NCBI Gene Expression Omnibus (GEO) database under accession code GSE210381 [https://www.ncbi.nlm.nih.gov/geo/].
ATAC-seq and ChIP-seq datasets used in this study were retrieved from public databases and a full list of files with accession is available in Supplementary Table S15.
Accession details to publicly available gene expression datasets used for Supplementary Figure S8 are mentioned in the figure legend.
All data supporting the findings of this study are available within the manuscript and supplementary information/Source Data file or from the corresponding author upon reasonable request.

Field-specific reporting

Please select the one below that is the best fit for your research. If you are not sure, read the appropriate sections before making your selection.

☒ Life sciences ☐ Behavioural & social sciences ☐ Ecological, evolutionary & environmental sciences

For a reference copy of the document with all sections, see nature.com/documents/nr-reporting-summary-flat.pdf

Life sciences study design

All studies must disclose on these points even when the disclosure is negative.

Sample size

GWAS: We analyzed 562 PA cases (223 women and 339 men) from the Hôpital Européen Georges Pompidou (HEGP) and 950 controls from the Paris Prospective Study III (PPS3) (311 women and 639 men) for ~680000 genotyped Single Nucleotide Polymorphisms (SNPs)
As this study deals with a disease representing approximately 5% of patients with arterial hypertension, but which is largely underdiagnosed, no samples sizes were calculated, but instead all available samples of patients with PA were included in the study. The number of subjects in the control population was calculated to represent at least a ~1.5x ratio over cases according to the available population matched by sex and age.
In vitro cell studies: 2 independent experiments performed in quaduplicate

Data exclusions

After genotyping, samples not fitting the quality control standards were excluded from further analyses.
Outliers on the first 2 components of the Principal component analysis plot (PLINK) were removed following classical procedures to obtain a homogenous population for the GWAS.
Individuals with a PI HAT (proportion of IBD) >0.125 with another individual were removed.

Replication

The results of the GWAS analysis in the discovery cohort were replicated in three different European cohorts and matched controls:
1) German cohort: 399 German PA patients (255 men and 144 women) and 1847 controls (KORA) were analyzed.
2) Italian cohort: 107 patients (66 men and 41 women) and 300 normotensive population controls (HYPERGENES) were analyzed.
3) Second French dataset, with 94 patients (59 men, 35 women) and 199 SUVIMAX controls.
The associations on chromosomes 1, 11 and 13 observed in the discovery cohort were replicated in the German cohort and in the replication meta-analysis including the three replication cohorts. Moreover they were confirmed by a global meta-analysis involving all case-control studies.
The associations on chromosome X as well as women-specific associations on chromosomes 1 and 13 were not replicated, which may be due to a slightly lower number of women among cases or to different ancestry-related linkage disequilibrium structures between the discovery and the replication cohorts.

In vitro cell studies: 2 independent experiments were performed in quaduplicate.

Randomization

Randomization is not relevant to this study, as this is a case-control GWAS study. For each cohort, the sex was included as a covariate in the logistic regression, as well as the first 10 components of the multi-dimensional scaling method applied on the identity-by-state matrix (or of the Principal Component Analysis) in order to account for potential population stratification.

Blinding

The investigators could not be blinded during data collection and analysis, as case and control cohorts originated from different protocols and/or required a thorough clinical investigation.

Reporting for specific materials, systems and methods

We require information from authors about some types of materials, experimental systems and methods used in many studies. Here, indicate whether each material, system or method listed is relevant to your study. If you are not sure if a list item applies to your research, read the appropriate section before selecting a response.

Materials & experimental systems

n/a	Involved in the study
☐	☒ Antibodies
☐	☒ Eukaryotic cell lines
☒	☐ Palaeontology and archaeology
☐	☒ Animals and other organisms
☐	☒ Human research participants
☒	☐ Clinical data
☒	☐ Dual use research of concern

Methods

n/a	Involved in the study
☒	☐ ChIP-seq
☒	☐ Flow cytometry
☒	☐ MRI-based neuroimaging

Antibodies

Antibodies used	Custom made anti-CYP11B2 (hCYP11B2-41-13C, mouse monoclonal) was kindly gifted from Prof. Celso Gomez-Sanchez, University of Missisipi: Celso E. Gomez-Sanchez - University of Mississippi Medical Center Cat# CYP11B2, RRID:AB_2650562<br>Anti-α-Tubulin antibody: Sigma-Aldrich Cat# T9026, RRID:AB_477593
Validation	Anti-α-Tubulin antibody used for Western blot was choosen because validated by the manufacturer (https://antibodyregistry.org/search.php?q=AB_477593) and already published elsewhere to be validated for such application in human (Colin et al, Biallelic variants in UBA5 reveal that disruption of the UFM1 cascade can result in early onset encephalopathy, Am J Hum Genet, 99(3):695-703 (2016)).<br>The validation of CYP11B2 antibody was described in Gomez-Sanchez et al, Development of monoclonal antibodies against human CYP11B1 and CYP11B2. Mol Cell Endocrinol. 5;383(1-2):111-7 (2014).<br>All these antibodies gave a specific band at the good molecular weight.

Eukaryotic cell lines

Policy information about cell lines

Cell line source(s)	The human adrenocortical carcinoma cell line H295R strain 2 (H295R-S2), kindly provided by W. E. Rainey, University of Michigan Ann Arbor, MI
Authentication	Wang, T. et al. Comparison of aldosterone production among human adrenocortical cell lines. Horm Metab Res 44, 245-50 (2012). Human adrenal cell lines SW13, CAR47, the NCI-H295 and its sub-strains and sub-clones were compared with regard to aldosterone production and aldosterone synthase (CYP11B2) expression. The results indicated that the H295R-S2 and the clonal cell lines, HAC13, HAC15 and HAC50 produced the highest levels of aldosterone and responded well to Angiotensin II.
Mycoplasma contamination	The cell lines tested negative for mycoplasma contamination.
Commonly misidentified lines<br>(See ICLAC register)	No commonly misidentified cell lines were used in the study

Animals and other organisms

Policy information about studies involving animals; ARRIVE guidelines recommended for reporting animal research

Laboratory animals	Adrenal tissues from four 12 weeks old male and female C57/BL6-SC129 mice.<br>Mice were housed in animal facilities on a 12 h light/dark cycle at temperature 18-22 degree and humidity 50-60%.
Wild animals	No wild animals were used in the study.
Field-collected samples	No field collected samples were used in the study.
Ethics oversight	Animal studies were conducted according to the guidelines formulated by the European Commission for experimental use (Directive 2010/63/EU) and were approved by the Institut National de la Santé et de la Recherche Médicale, by the local Ethics committee of Paris Descartes University (N°17-020) and by the French Ministère de l'Enseignement Supérieur, de la Recherche et de l'Innovation.

Note that full information on the approval of the study protocol must also be provided in the manuscript.

Human research participants

Policy information about studies involving human research participants

Population characteristics

French cohorts: 562 PA cases (223 women and 339 men; mean age 61.6 ± 13.2 years-old, 321 with aldosterone producing adenoma and 240 with bileteral adrenal hyperplasia) as well as a second set of 94 cases (59 men and 35 women, mean age 51.7 ± 9.7 years old, 64 with aldosterone producing adenoma and 30 with bilateral adrenal hyperplasia) were analyzed in the present study. Methods for screening and subtype identification of PA were performed according to institutional and the Endocrine Society guidelines (Funder, J.W. et al. J Clin Endocrinol Metab 101, 1889-916, 2016; Amar, L. et al. Ann Endocrinol (Paris) 77, 179-86, 2016; Baron, S. et al. J Hypertens 36, 1592-1601, 2018). For a subset of 122 patients, somatic mutation analysis performed on fresh frozen APA tissue by whole exome or Sanger sequencing was available (50 KCNJ5, 23 CACNA1D, 7 ATP1A1, 4 ATP2B3, 5 CTNNB1, 1 APC, 32 negative 56. For an additional subset of 42 patients with APA included in the GWAS, detailed genetic analysis by CYP11B2-immunohistochemistry guided next generation sequencing, histological information and steroid profiles were available (18 KCNJ5, 11 CACNA1D, 6 ATP1A1, 4 ATP2B3, 3 negative).

German cohort: 399 German PA patients (255 men and 144 women, mean age 59.4 ± 12.3 years-old, 214 with aldosterone producing adenoma and 135 with bilateral adrenal hyperplasia) were analyzed. The diagnosis of PA was made according to the Endocrine Society Practice Guidelines (Funder, J.W. et al. J Clin Endocrinol Metab 101, 1889-916, 2016). 127 patients had genetic analysis performed either by whole exome or Sanger sequencing. Mutation status was the following: 56 KCNJ5, 14 CACNA1D, 9 ATP1A1, 14 ATP2B3, 2 CTNNB1, 32 negative.

Italian cohort: 107 patients with PA (66 men and 41 women, mean age 59.3±11.5 years-old, 76 with aldosterone producing adenoma and 31 with bilateral adrenal hyperplasia) were referred to the European Society of Hypertension Specialized Center of Excellence of the University of Padua, Italy. They were submitted to subtype identification by adrenal vein sampling without stimulation and the diagnosis was performed following the PAPY Study (Rossi, G.P. et al. J Am Coll Cardiol 48, 2293-300, 2006). and the Endocrine Society guidelines (Funder, J.W. et al. J Clin Endocrinol Metab 101, 1889-916, 2016). 79 patients had genetic analysis performed by targeted Sanger sequencing and 20 KCNJ5 and 3 CACNA1D mutations were identified.

The Paris Prospective Study 3 (PPS3) population characteristics are described in :
Empana, J.P. et al. Paris Prospective Study III: a study of novel heart rate parameters, baroreflex sensitivity and risk of sudden death. Eur J Epidemiol 26, 887-92 (2011); Proust, C. et al. Contribution of Rare and Common Genetic Variants to Plasma Lipid Levels and Carotid Stiffness and Geometry: A Substudy of the Paris Prospective Study 3. Circ Cardiovasc Genet 8, 628-36 (2015). For the present study, 950 subjects were analysed (639 men and 311 women, mean age 65.3±6.4 years-old).

SUVIMAX participants characteristics are described in Hercberg, S. et al. The SU.VI.MAX Study: a randomized, placebo-controlled trial of the health effects of antioxidant vitamins and minerals. Arch Intern Med 164, 2335-42 (2004). In this study, 199 subjects were analysed (126 men and 73 women, mean age 50.4±5.9 years-old).

The Cooperative Health Research in the Region of Augsburg (KORA) cohort characteristics are described in Wichmann, H.E., Gieger, C., Illig, T. & Group, M.K.S. KORA-gen--resource for population genetics, controls and a broad spectrum of disease phenotypes. Gesundheitswesen 67 Suppl 1, S26-30 (2005). In the present study 1847 subjects were analysed (915 men and 932 women, mean age 46.22±11.92 years-old).

The characteristics of participants of HYPERGENES cohort were described in Salvi, E. et al. Genomewide association study using a high-density single nucleotide polymorphism array and case-control design identifies a novel essential hypertension susceptibility locus in the promoter region of endothelial NO synthase. Hypertension 59, 248-55 (2012). In the present study 300 subjects were included (186 men and 114 women, mean age 60.9± 6.5 years-old).

Recruitment

Patients:
French cohort: Patients with PA were recruited within the COMETE (COrtico- et MEdullo-surrénale, les Tumeurs Endocrines) network or in the context of genetic screening for familial hyperaldosteronism at the Genetics department of the HEGP. Methods for screening and subtype identification of PA were performed according to institutional and the Endocrine Society guidelines. In patients diagnosed with primary aldosteronism, a thin slice CT scan or MRI of the adrenal and/or an adrenal venous sampling (AVS) were performed to differentiate between unilateral and bilateral aldosterone hypersecretion. All patients gave written informed consent for genetic and clinical investigation. Procedures were in accordance with institutional guidelines.

German cohort: Patients with PA were recruited at the Munich center of the Else Kröner-Fresenius HyperaldosteroinismusRegister - German Conn Registry. The diagnosis of PA was made according to the Endocrine Society Practice Guidelines. The screening test consisted of a baseline plasma aldosterone-to-renin ratio (ARR; cut-off 12.0ng/U, sitting position). If elevated, diagnosis of PA was confirmed by an abnormal confirmatory test. The subtype diagnosis between unilateral and bilateral adrenal hyperplasia was based on simultanous bilateral adrenal vein sampling without ACTH stimulation.

Italian cohort: Patients with PA were referred to the European Society of Hypertension Specialized Center of Excellence of the University of Padua, Italy. They underwent a biochemical screening for secondary causes of hypertension and provided informed written consent (Prot.1925P/2009, Comitato Etico per la Sperimentazione, Azienda Ospedaliera di Padova, Regione Veneto). They were submitted to subtype identification by adrenal vein sampling without stimulation and the diagnosis was performed following the PAPY Study and the Endocrine Society guidelines

Controls:
The Paris Prospective Study 3 (PPS3) is an observational prospective study evaluating the role of a set of novel biomarkers on cardiovascular disease in a healthy population. Briefly, the PPS3 cohort consists of 10157 volunteers aged 50 to 75 years recruited from a large preventative medical center, the Centre d'Investigations Préventives et Cliniques in Paris (France) between June 2008 and May 2012.

Participants of the SUVIMAX study were healthy volunteers free of hypertension, cardiovascular disease, or cancer at baseline, recruited in metropolitan France and of European descent. Genetic information was available in a subsample of 1518 participants.

The Cooperative Health Research in the Region of Augsburg (KORA) cohort comprises several population-based cohort studies in the region of Augsburg, Southern Germany. KORA S3 is an independent population-based sample aged 25 to 74 years that was studied between 1994 and 1995. As controls we selected 1847 subjects, who participated in a follow-up examination of S3 (KORA F3, 2004 - 2005) and were then persons between the ages of 35 and 84 years.

A random sample of 300 Italian normotensive controls of the HYPERGENES cohort were included. A participant could be included in HYPERGENES as normotensive if he/she could self report to be of Caucasian Origin, was unrelated with other participants, had DBP <85 mmHg and SBP <135 at least until 55 years of age and had never been treated for hypertension.

Ethics oversight

French cohort: Patients with PA were recruited within the COMETE (COrtico- et MEdullo-surrénale, les Tumeurs Endocrines) network (COMETE-HEGP protocol, authorization CPP Ile de France 2012-A00508-35) or in the context of genetic screening for familial hyperaldosteronism at the Genetics department of the HEGP. All patients gave written informed consent for genetic and clinical investigation. Procedures were in accordance with institutional guidelines.

German cohort: All subjects gave written informed consent for genetic investigation (University Hospital Munich protocol 379-10, Ethikkommission der LMU München)

Italian cohort: Patients with PA (APA and BAH) were referred to the European Society of Hypertension Specialized Center of Excellence of the University of Padua, Italy. They underwent a biochemical screening for secondary causes of hypertension and provided informed written consent (Prot.1925P/2009 Comitato Etico per la Sperimentazione, Azienda Ospedaliera di Padova, Regione Veneto).

The Paris Prospective Study 3 (PPS3): All participants have provided written informed consent and the study protocol was approved by the Ethics Committee of the Cochin Hospital (Paris). The study is registered in the international trial registry (URL: http://www.clinicaltrials.gov. Unique identifier: NCT00741728).

SUVIMAX ethics oversights are described in Hercberg, S. et al. The SU.VI.MAX Study: a randomized, placebo-controlled trial of the health effects of antioxidant vitamins and minerals. Arch Intern Med 164, 2335-42 (2004).

The Cooperative Health Research in the Region of Augsburg (KORA) ethics oversights are described in Wichmann, H.E., Gieger, C., Illig, T. & Group, M.K.S. KORA-gen--resource for population genetics, controls and a broad spectrum of disease phenotypes. Gesundheitswesen 67 Suppl 1, S26-30 (2005).

HYPERGENES cohort ethics oversights were described in Salvi, E. et al. Genomewide association study using a high-density single nucleotide polymorphism array and case-control design identifies a novel essential hypertension susceptibility locus in the promoter region of endothelial NO synthase. Hypertension 59, 248-55 (2012).

Note that full information on the approval of the study protocol must also be provided in the manuscript.
